# Walking and Balance Outcomes Are Improved Following Brief Intensive Locomotor Skill Training but Are Not Augmented by Transcranial Direct Current Stimulation in Persons With Chronic Spinal Cord Injury

**DOI:** 10.3389/fnhum.2022.849297

**Published:** 2022-05-11

**Authors:** Nicholas H. Evans, Cazmon Suri, Edelle C. Field-Fote

**Affiliations:** ^1^Shepherd Center, Crawford Research Institute, Atlanta, GA, United States; ^2^Department of Applied Physiology, Georgia Institute of Technology, Atlanta, GA, United States; ^3^Department of Rehabilitation Medicine, Emory University School of Medicine, Atlanta, GA, United States

**Keywords:** anodal-tDCS, exercise intensity, walking, spinal cord injury (SCI), motor learning

## Abstract

Motor training to improve walking and balance function is a common aspect of rehabilitation following motor-incomplete spinal cord injury (MISCI). Evidence suggests that moderate- to high-intensity exercise facilitates neuroplastic mechanisms that support motor skill acquisition and learning. Furthermore, enhancing corticospinal drive via transcranial direct current stimulation (tDCS) may augment the effects of motor training. In this pilot study, we investigated whether a brief moderate-intensity locomotor-related motor skill training (MST) circuit, with and without tDCS, improved walking and balance outcomes in persons with MISCI. In addition, we examined potential differences between within-day (online) and between-day (offline) effects of MST. Twenty-six adults with chronic MISCI, who had some walking ability, were enrolled in a 5-day double-blind, randomized study with a 3-day intervention period. Participants were assigned to an intensive locomotor MST circuit and concurrent application of either sham tDCS (MST+tDCS_sham_) or active tDCS (MST+tDCS). The primary outcome was overground walking speed measured during the 10-meter walk test. Secondary outcomes included spatiotemporal gait characteristics (cadence and stride length), peak trailing limb angle (TLA), intralimb coordination (ACC), the Berg Balance Scale (BBS), and the Falls Efficacy Scale-International (FES-I) questionnaire. Analyses revealed a significant effect of the MST circuit, with improvements in walking speed, cadence, bilateral stride length, stronger limb TLA, weaker limb ACC, BBS, and FES-I observed in both the MST+tDCS_sham_ and MST+tDCS groups. No differences in outcomes were observed between groups. Between-day change accounted for a greater percentage of the overall change in walking outcomes. In persons with MISCI, brief intensive MST involving a circuit of ballistic, cyclic locomotor-related skill activities improved walking outcomes, and selected strength and balance outcomes; however, concurrent application of tDCS did not further enhance the effects of MST.

**Clinical Trial Registration:**

[ClinicalTrials.gov], identifier [NCT03237234].

## Introduction

Recovery of motor function, including walking and balance, is central to rehabilitation for persons with motor-incomplete spinal cord injury (MISCI). Given the functional and health-related value of standing and walking (Olson et al., 2018), even small improvements in locomotor function can have a significant impact on the long-term health, quality of life, and community re-integration of people living with MISCI (Hiremath et al., 2017; Gasper et al., 2019; McMillan et al., 2021). Because resources often limit the amount of time that persons with SCI have access to rehabilitation services, therapies that extract the greatest benefit in the least amount of time are of ongoing interest. Further, because ongoing practice is important for maintaining gains acquired during rehabilitation, there is value in examining training approaches that could feasibly be carried out with supervision in the home or a community setting once individuals are discharged from traditional rehabilitation.

The restoration of motor function after neurological injury is dependent on the extent to which motor learning (i.e., the acquisition and long-term retention of new or previously learned motor skills) can be optimized. Frameworks for conceptualizing motor skill acquisition and retention have been described extensively (Schmidt, 1975; Higgins, 1991). Existing evidence indicates that training-induced motor learning is achieved through a combination of online (within-day) and offline (between-day) processes (Robertson et al., 2004; Reis et al., 2009; Dayan and Cohen, 2011). The mechanisms underlying performance improvements observed within minutes to hours of a single training session (online learning) are different from those observed in the day(s) between training sessions (offline learning) (El-Sayes et al., 2019; Wanner et al., 2020). Various interventions have been deployed with the aim of specifically targeting mechanisms that support motor skill acquisition and retention. Intensive exercise (Singh et al., 2014a; Skriver et al., 2014) and non-invasive brain stimulation (Nitsche et al., 2003; Gomes-Osman and Field-Fote, 2015) have been explored as viable neuromodulation approaches that have the capacity to reinforce mechanisms that subserve neuroplasticity and motor learning through both online and offline processes.

Recent findings emphasize the importance of training *intensity* as a means of improving motor function and influencing neuroplasticity in persons with neuropathology (Fisher et al., 2008; Mang et al., 2013; Leech et al., 2016; Holleran et al., 2018). Of particular interest is the intensity-dependent release of brain-derived neurotrophic factor (BDNF), which facilitates and supports neuroplastic events within the nervous system (Yoshi and Constantine-Paton, 2009; McDonnell et al., 2013; Guo et al., 2014, 2018; Mang et al., 2014; Phillips et al., 2014; Skriver et al., 2014; Dinoff et al., 2016; Cobianchi et al., 2017; Inoue et al., 2018). Exogenous delivery of BDNF is associated with synaptic strengthening, neuronal sprouting, and improved locomotor recovery in pre-clinical models of SCI (Jakeman et al., 1998). Moderate- to high-intensity exercise is associated with increased endogenous levels of BDNF in persons with SCI (Rojas Vega et al., 2008; Leech and Hornby, 2017). In neurologically intact adults, a single session of intense cycling is associated with an increase in serum BDNF (Skriver et al., 2014) and enhanced motor cortical activity (Holman and Staines, 2021), which, in both cases, were positively correlated with retention of a novel motor task. It is believed that higher-intensity motor training is superior to lower-intensity training for improving motor function through the upregulation and release of BDNF that encourages mechanisms that support skill acquisition and learning.

Transcranial direct current stimulation (tDCS) is increasingly used in rehabilitation research as a neuromodulatory approach to influence excitability of cortical (Nitsche and Paulus, 2000) and cerebellar (Celnik, 2015) networks. Most often, the aim is to “prime” neural circuits to increase corticospinal activation and to augment effects of motor skill training (Sriraman et al., 2014; Page et al., 2015). Evidence in non-injured adults indicates that concurrent application of anodal-tDCS over the primary motor cortex with motor skill training enhances learning by facilitating offline mechanisms that support motor program consolidation (Reis et al., 2009; Kim et al., 2021b). Furthermore, cathodal-tDCS over the cerebellum reduces the inhibitory influence of the cerebellum on thalamo-cortical structures involved in motor control (Galea et al., 2009) and is associated with improved locomotor and balance function in persons with neuropathology (Kaski et al., 2013).

Several prior studies by our lab and others have investigated tDCS for augmenting upper extremity training in persons with tetraplegia (Gomes-Osman and Field-Fote, 2015; Yozbatiran et al., 2016; Cortes et al., 2017; Estes et al., 2017; Potter-Baker et al., 2018). Very little is known about the influence of combining tDCS with motor training to improve lower extremity motor function in persons with MISCI. To date, only two studies have examined this combinatorial intervention approach for improving locomotor and balance function. Both involved the application of tDCS along with robotic-assisted treadmill training (Kumru et al., 2016; Raithatha et al., 2016), which is a locomotor training approach that often lacks intensity (Kressler et al., 2013) and is rarely accessible to persons with MISCI once discharged to the home. Moreover, neither study examined the acute temporal influence (i.e., within- and between-day responses) associated with these combined interventions. Characterizing online vs. offline training effects in persons with MISCI will be valuable for future studies aimed at exploring the potential mechanisms underlying motor learning following combined motor training and tDCS.

In light of the above, locomotor skill training that capitalizes on the neuroplastic effects of intensive exercise and can be implemented in the home or community may have potential for meaningful impact on the long-term restoration and retention of walking and balance function. Additionally, training effects may be augmented by tDCS by influencing mechanisms that support motor skill acquisition and consolidation. Previously, we reported significant persistent effects of 3 days of intensive locomotor skill training on measures of walking ability (Evans and Field-Fote, 2022). The purpose of this study was to examine the within-day and between-day effects of three consecutive days of moderate-intensity motor skill training (MST), with and without tDCS, on measures of walking and balance function in persons with MISCI. We hypothesized that MST would be associated with improvements in walking and balance function and that the addition of tDCS would lead to greater improvements than MST alone.

## Materials and Methods

### Study Design

**FIGURE 1:**
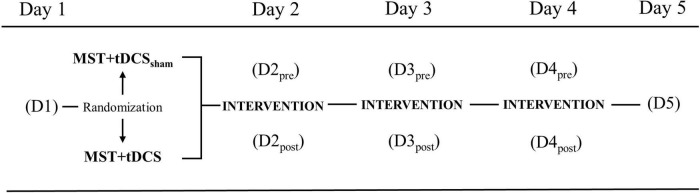
Study design with outcomes collected at baseline Day-1 (D1), pre-/post-intervention on Day-2 (D2), Day-3 (D3), Day-4 (D4), and 24-h post-intervention on Day-5 (D5). *MST+tDCS_*sham*_*, motor skill training plus sham transcranial direct current stimulation; *MST+tDCS*, motor skill training plus active transcranial direct current stimulation.

This pilot study was conducted as a multi-session double-blind, randomized intervention (ClinicalTrials.gov Identifier: NCT03237234). Participants were randomly assigned to one of two groups ([1] MST with concurrent sham tDCS [MST+tDCS_sham_] or [2] MST with concurrent active tDCS [MST+tDCS]) using the REDCap randomization module. Participants, trainers, and assessors were blinded to tDCS group allocation. The tDCS was applied by a staff member not otherwise involved in the study. The study was carried-out over five consecutive days (Monday-Friday), with three intervention days (Tuesday–Thursday). The 3-day intervention was selected to establish preliminary evidence of efficacy prior to undertaking a longer study. Outcomes were assessed at baseline on Day-1 (D1) and 24-h post-intervention on Day-5 (D5) to examine cumulative and persistent effects of intervention. To examine within- and between-day effects of intervention on outcome measures associated with walking, a subset of selected outcomes were assessed pre- (D2_pre_, D3_pre_, D4_pre_) and post-intervention (D2_post_, D3_post_, D4_post_) on each intervention day (Figure 1).

### Study Sample

The sample size was calculated on the basis of change in the primary outcome measure (overground walking speed) previously reported in participants with MISCI, wherein an effect size of 0.69 was identified (Manella et al., 2013). To achieve a power = 0.80 at α = 0.10 (one-sided), a sample size of 15 participants per group was calculated (G*Power 3.1: F tests, ANOVA repeated measures, between-factors). Inclusion criteria were: (a) chronic MISCI (≥12 months) at/above the neurological level T10, (b) aged 18–70 years, (c) able to stand for ≥5 min, and (d) able to advance each leg independently ≥3 steps. Exclusion criteria were: (a) progressive spinal lesions, (b) uncontrolled cardiorespiratory conditions, (c) altered cognitive status, (d) orthopedic pathology, (e) intracranial metal, and (f) history of seizures. Injury characteristics [i.e., ASIA Impairment Scale (AIS) classification and lower extremity motor scores (LEMS)] were obtained at the time of enrollment from participant medical records (if completed within the prior 6-months) or following neurological examination by a member of the research team.

### Interventions

#### Motor Skill Training

**FIGURE 2:**
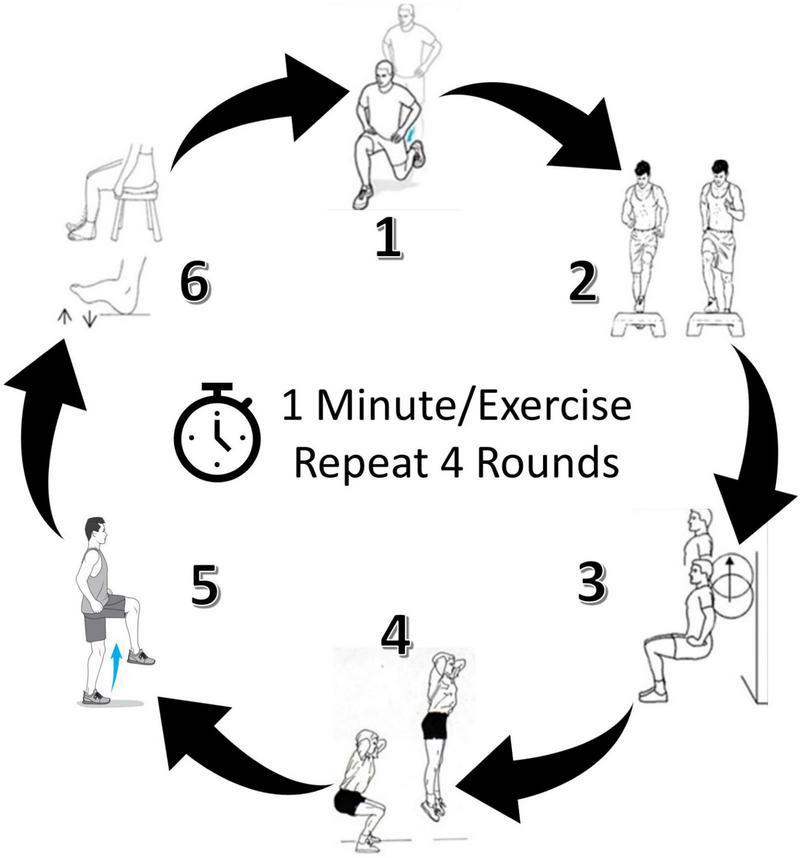
Locomotor-related motor skill training (MST) circuit. Six exercises were performed for one minute each, and the circuit was completed four times. Target MST intensity was 40–59% of heart rate reserve. Images of a representative participant completing the circuit can be found elsewhere (Evans and Field-Fote, 2022).

Details of the MST intervention have been described previously (Evans and Field-Fote, 2022). Briefly, six activities were selected that could feasibly be performed (with supervision) in the home or a wellness setting, with the aim of targeting muscle groups and movement patterns important to walking ability in persons with SCI (Crozier et al., 1992; Kim et al., 2004; Wirth et al., 2008a; van Middendorp et al., 2011). Each motor task was performed for 60 seconds in consecutive order and repeated 4 times as a circuit (Figure 2). Participants were asked to complete as many repetitions as possible in the time allotted for each activity. The MST activities were intended to challenge upright standing balance and promote rapid volitional activation and deactivation of lower extremity muscles (i.e., hip, knee, and ankle extensors/flexors) through movements characterized by cyclic (Vietnen and Welch, 2020) and/or ballistic (Cordner et al., 2021) sequences. One seated activity was included in the circuit to provide an opportunity for active rest. For participants with greater motor impairment, modifications that did not substantively alter the intent of the activity were provided to ensure that all participants could complete each task. Examples included: providing a fixed bar for upper extremity support in cases where balance deficits made completing an activity unsafe or impossible (e.g., attempting a ballistic jump during activity #4); and providing manual assistance in cases where participants lacked sufficient strength to achieve the range of motion needed to accomplish the goal of the task (e.g., lifting the foot to the step during activity #2).

Training intensity was monitored continuously throughout the MST circuit and quantified via heart rate obtained using a chest-worn monitor and wristwatch (Polar FT1; Polar Electro Inc., Woodbury, NY, United States). Average (HR_avg_) and peak (HR_peak_) heart rate were recorded for each session. During MST, participants were encouraged to perform the activities as rapidly as possible with the intent to maintain a moderate exercise intensity, recognized as 40–60% heart rate reserve (HRR) (ACSM et al., 2018). Ranges for %HRR were established for each participant according to resting heart rate and HR_peak_ obtained from baseline maximal graded exercise testing performed via upper body cycle ergometer (Monark 881E Arm Ergometer; Monark Exercise AB, Vansbro, Sweden). Graded exercise testing procedures followed accepted practices (ACSM et al., 2018).

#### Transcranial Direct Current Stimulation

TDCS (ActivaDose II; Activa Tek Inc., Gilroy, CA, United States) was delivered via two 5 × 5 cm electrodes (0.9% saline-soaked sponges): the anode was placed slightly anterior to the vertex over bilateral primary motor cortices, and the cathode was placed at the inion over the cerebellum. This electrode montage was chosen based on previous reports for modulation of brain regions involved in lower extremity motor control (Kaski et al., 2012, 2013; Mang et al., 2016). Electrodes were secured using elastic straps that had been marked to replicate placement location. For participants in the active tDCS condition, 20-min of stimulation was delivered concurrently with MST at an intensity of 2 mA (current density = 0.80 A/m^2^; charge density = 0.96 kC/m^2^). For participants receiving sham tDCS, set-up procedures were identical to the active tDCS condition; however, a ramp-up/ramp-down sequence was employed wherein stimulation was gradually increased to 2 mA and then decreased to 0 mA over approximately 40-s at the start of each MST session. Study personnel involved in administering tDCS were not involved in any other aspect of the study, and those involved in MST delivery and data collection were blinded to the stimulation condition.

### Outcome Measures

#### Walking Measures

The primary outcome measure of walking ability was overground walking speed (m/s), which is a common clinical measure and is predictive of community independence among persons with SCI (van Hedel and Group, 2006; van Silfhout et al., 2017). Measures of walking speed were obtained during the 10-meter walk test (10MWT). At each time point, participants completed three, 10 MWT trials over a 14-m path with 2-meter acceleration and deceleration zones located at the beginning and end of the path. Walk trials were separated by 2-min of seated rest. Instructions provided prior to each walk were to “*walk as quickly and as safely as possible.*” Participants were permitted to use their usual assistive devices, which included rolling walkers (*n* = 10), crutches/canes (*n* = 6), ankle-foot orthoses (*n* = 3), and a Swedish knee cage (*n* = 1). Assistive walking devices were kept consistent between all walk trials. Mean walking speed of the three walk trials at each time point was used in the analysis.

Secondary outcome measures of walking ability included kinematic measures of gait quality. Kinematic data were obtained during each 10MWT using a 3D inertial measurement unit (IMU) motion capture system (Xsens MVN Biomech Awinda; Xsens Technologies BV, Enschede, NL) and a 7-meter-long instrumented walkway (GAITRite; CIR Systems Inc., Sparta, NJ, United States). Body dimensions were measured, and IMU sensors were affixed by elastic straps to the head, sternum, and pelvis, and bilaterally to the hands, forearms, upper arms, shoulders, thighs, shanks, and feet in accordance with manufacturer’s specifications. IMU calibration was performed with participants standing in the fundamental position (N-pose; with arms by their sides and without the use of an assistive device). Kinematic data were sampled at 60 hz for each walk trial. A customized computer code was developed in MATLAB (version R2021a; The MathWorks Inc., Natick, MA, United States) to extract relevant kinematic data generated by the IMU system (version 2019.0.0; Xsens MVN, Enschede, NL).

Stride frequency (i.e., cadence) and stride length are coupled to walking speed modulation in persons with SCI (Pepin et al., 2003) and therefore, were considered outcomes of interest. Stride frequency (strides/min) and stride length (cm) of the stronger and weaker lower limbs were extracted for each walk trial from full steps registered along the length of the instrumented walkway. Data from partial steps acquired at the beginning and the end of the instrumented walkway were excluded, and the average value across the three walk trials was used in the analysis. Outcomes extracted from IMU data included the peak trailing limb angle (TLA [°]) and the angular component of the coefficient of correspondence (ACC). Propulsive force is diminished in persons with SCI and is associated with a reduction in overground walking speed (Peters et al., 2018). Sixty-five percent of the increase in propulsive force generated during walking speed modulation can be attributed to increases in the TLA (Hsiao et al., 2015). Peak TLA has been characterized and validated as a surrogate measure of propulsive force in healthy adults (Hsiao et al., 2015) as well as in persons with stroke (Lewek and Sawicki, 2019). We examined the extent to which the TLA of the stronger and weaker limbs was amenable to intervention. MATLAB output for TLA quantification was validated against TLA data obtained from a non-injured test participant using a 3D optical motion capture system (Vicon Motion Systems Ltd., United Kingdom) previously used to quantify the TLA (Miyazaki et al., 2019). Briefly, the positions of anatomical landmarks in the global frame were determined beginning at the pelvis and moving distally to the lower extremities and feet using a link segment (kinematic chain) model. For both the left and right lower extremities, TLA was calculated from sagittal plane kinematics according to the angle created by the vertical line passing through the hip and ankle joint during IMU calibration and the line connecting the location of the hip and ankle joint during each stride (Miyazaki et al., 2019). TLA values obtained during the acceleration and deceleration periods of each walk trial were excluded by calculating peak TLA from the average value obtained during the middle 50% of strides. The average peak TLA (for each lower limb) from all walk trials at each time point was used in the analysis.

Intralimb coordination of the weaker and stronger limbs was calculated according to the angular component of the coefficient of correspondence (ACC) based on methods previously developed by members of our team (Tepavac and Field-Fote, 2001). The ACC indicates the degree of stride cycle-to-cycle variability in hip-knee relative motion plots. Prior investigations have used this approach to examine intralimb coordination as a measure of the integrated function of motor systems involved in cyclic locomotor behavior in persons with SCI (Field-Fote and Tepavac, 2002; Nooijen et al., 2009; Awai and Curt, 2014; Holleran et al., 2018). In keeping with these reports, we quantified the consistency of cycle-to-cycle kinematics of the hip and knee joints. Similar to TLA calculations, a single ACC value for each lower limb was computed from the middle 50% of strides for each walk trial, and the average ACC of three walks at each time point was used in the analysis. An ACC value of “1” indicates perfect cycle-to-cycle hip-knee angle consistency and a value of “0” indicates no cycle-to-cycle consistency. Stronger and weaker limbs identified for stride length, TLA, and ACC were determined from lower extremity motor scores obtained from manual muscle tests at baseline (D1). All walking measures were collected at all-time points over the course of the study.

#### Balance Function

Balance performance and mobility confidence were assessed at baseline and at 24-h post-intervention using the Berg Balance Scale (BBS) (Lemay and Nadeau, 2010) and the Falls Efficacy Scale-International (FES-I) (Dewan and MacDermid, 2014), respectively. The BBS contains 14 functional test items with each item scored on a 0–4 scale, with “0” indicating the lowest level of function and “4” indicating the highest level of function. Higher scores on the BBS indicate better balance performance. In persons with SCI, concern for falling limits individual performance during overground motor tasks irrespective of functional ability to perform the task (John et al., 2010) and may influence balance performance. Fear of falling was assessed using the FES-I 16-item questionnaire. Each question addresses the participant’s concern for falling during specific activities of daily living. Participants were asked to rate their concern for falling on each item using a four-point scale, where “1” indicates “*Not at all concerned*” and “4” indicates “*Very concerned.*” Higher scores on the FES-I indicate a greater fear of falling. To minimize participant burden, the BBS and the FES-I were only performed at baseline (D1) and 24-h post-intervention (D5). Total scores for both measures were used in the analysis.

### Data Analysis

Data were managed in Microsoft Excel and analyzed using SPSS v27 (IBM Corp, 2020). Outcomes were examined for outliers and distributional abnormalities. One participant in the MST+tDCS group did not complete D4_post_ testing. In this case, mean replacement for the D4_post_ time point was used based on the average of the previous two post-intervention time points. Mean group-level and full sample training characteristics (i.e., total training duration, training intensity) were calculated by first recording the mean total time and %HRR for each participant at each time point and then taking the average of these values across all intervention days (i.e., [D2 mean value + D3 mean value + D4 mean value]/3). The effects of intervention on walking speed, cadence, stride length, TLA, BBS, and FES-I were examined using a linear mixed-effects model with TIME, GROUP, and TIME × GROUP interaction treated as fixed effects. SUBJECT was identified as a random factor using a “random intercepts by participant” approach. Covariance structure was modeled using variance components, with α set *a priori* at 0.10. An alpha of 0.10 was selected based on recommendations for designing and implementing pilot studies in clinical research (Moore et al., 2011), where higher levels of type I error rates (e.g., 10–25%) are accepted in order to screen for potential efficacious treatments and to avoid falsely rejecting interventions that may be beneficial. Model parameters were calculated using restricted maximum likelihood estimation (for small sample sizes; Meteyard and Davies, 2020). Degrees of freedom estimation was performed using Satterthwaite approximation. Given that participants completed multiple walk trials at each time point and to rule out the possible influence of walking speed variance on outcomes, we calculated the SD of the 3 walking speeds obtained from each participant at each time point. Linear mixed-effects model analysis revealed no differences in walk speed variance across TIME, *F*(7, 182) = 1.16, *p* = 0.327, or between GROUPs, *F*(1, 182) = 3.21, *p* = 0.075.

In the presence of significant findings for the primary and secondary outcome measures, post hoc pairwise comparisons examining differences in outcomes between time points were performed using paired-samples *t*-tests. In keeping with concern for falsely rejecting possible beneficial treatments, and given the small sample size of our study, we followed recommendations for developing exploratory and early phase studies (Feise, 2002; Parker and Weir, 2020) in which adjustment for multiple comparisons are not advised in order to decrease the chance of committing type II errors and to overcome the need to increase the sample size, which was not possible due to early termination of the study due to COVID-19 restrictions.

To examine within-day and between-day responses to intervention, cumulative change in outcomes for all within-day and between-day time points were calculated and between-groups and full sample differences were compared using independent samples *t*-test and paired samples *t*-test, respectively. ACC data for the stronger and weaker limbs were negatively skewed with significant Shapiro-Wilk’s tests (*p* < 0.05) at all-time points; therefore, ACC data were analyzed using nonparametric tests. Differences within stronger and weaker limb ACC over TIME were analyzed using the Friedman test. GROUP differences in change for the stronger and weaker limb ACC were examined using the Mann-Whitney *U*-test. *Post hoc* comparisons of differences between time points were examined using a Wilcoxon signed-rank test. Descriptive statistics for parametric analyses are reported as mean (SD) and for nonparametric tests are reported as median (IQR). Responsiveness of the primary outcome (overground walking speed) to intervention was assessed via *Cohen’s d_*z*_* (Lakens, 2013) according to criteria established for effect size (*ES*) interpretation in multicomponent rehabilitation interventions (Kinney et al., 2020), where *ES* was considered small (*d* = 0.14), medium (*d* = 0.31), or large (*d* = 0.55).

## Results

### Participants

**FIGURE 3:**
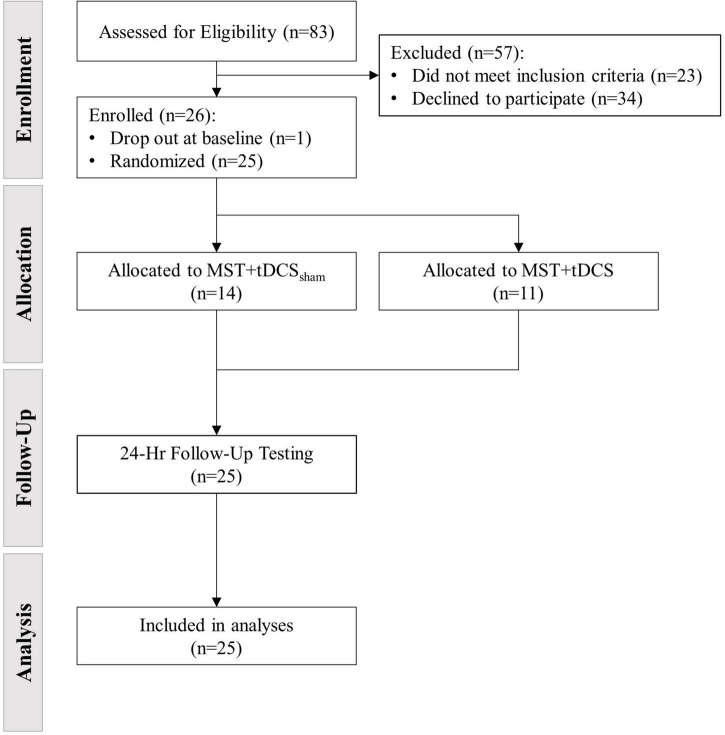
Recruitment, enrollment, and group allocation flow diagram.

Twenty-six participants with chronic, MISCI were enrolled in the study between *March*, *2017* to *March*, *2020*, with one withdrawal after baseline testing. The intended sample size of 30 participants (15/group) was not reached due to early termination of enrollment. Two protocol deviations (2 participants enrolled at 11-months post-injury) were considered necessary to maximize our recruitment targets. Randomization resulted in 14 participants allocated to the MST+tDCS_sham_ group and 11 participants allocated to the MST+tDCS group (Figure 3). Individual participant characteristics at baseline are presented in Table 1. Documented adverse events during the study have been reported previously (Evans and Field-Fote, 2022) and included cases of mild-to-moderate headache following tDCS and delayed-onset muscle soreness following MST. There were no between-groups differences in MST duration, *t*(23) = −0.13, *p* = 0.90, or intensity, *t*(23) = −0.25, *p* = 0.80. Mean MST duration and intensity for the full study sample was 37 min (*SD* = 6.1) and 51.9% HRR (*SD* = 14.3), respectively. Individual and group mean training duration at each intervention time point have been reported previously (Evans and Field-Fote, 2022).

**TABLE 1:** Individual participant characteristics according to intervention group at baseline.

Intervention group	Participant	Sex	Age	BMI	TSI	NLI	AIS	LEMS	GXT HR_peak_	GXT VO_2peak_	Walking speed (m/s)	Antispa smodics
MST + tDCS_sham_	01	F	19	19.6	12	C7	D	40	141	18.9	1.27	Yes
	02	M	37	22.4	228	C7	D	45	151	27.5	1.43	Yes
	03	M	55	26.6	12	C5	D	35	134	24.5	0.20	No
	04	M	58	19.4	46	C5	D	48	107	15.1	1.32	Yes
	05	F	33	18.6	115	C4	D	28	116	14.0	0.66	Yes
	06	M	56	27.2	98	C5	D	49	180	24.5	1.84	Yes
	07	M	20	26.6	24	C5	D	30	153	21.8	0.31	Yes
	08	M	50	23.3	236	C5	D	43	112	11.4	0.18	No
	09	M	60	29.9	35	C5	D	47	152	11.2	0.82	No
	10	M	54	31.1	12	C4	D	31	115	13.6	0.50	Yes
	11	M	51	25.5	32	C4	D	32	110	12.0	0.36	Yes
	12	F	63	18.6	65	T8	D	25	145	22.0	0.30	Yes
	13	M	36	21.3	202	C6	D	22	153	18.4	0.47	Yes
	14	F	62	15.8	188	C4	C	45	110	10.3	0.47	No
	**Mean** (***SD*)**	**N/A**	**46.7** **(15.0)**	**23.3** **(4.6)**	**93.2** **(85.3)**	**N/A**	**N/A**	**37.1** **(9.2)**	**134.2** **(22.7)**	**17.5** **(5.8)**	**0.72** **(0.53)**	**N/A**
MST + tDCS	01	F	48	20.6	42	T6	D	39	127	22.9	0.62	Yes
	02	M	44	25.7	246	C6	D	36	124	24.8	0.20	Yes
	03	M	50	19.3	276	C5	D	30	125	14.7	0.07	Yes
	04	M	49	30.4	31	C4	D	50	167	21.7	1.77	Yes
	05	M	45	25.9	22	C4	C	26	112	16.2	0.87	Yes
	06	F	29	19.8	11	T8	D	42	154	14.7	0.73	No
	07	M	47	39.6	11	C4	D	49	101	7.3	0.86	Yes
	08	M	69	27.8	24	C4	D	47	143	13.9	0.45	Yes
	09	M	51	30.9	75	C4	D	36	123	13.8	0.07	Yes
	10	M	64	25.1	66	C7	D	48	115	15.7	0.34	Yes
	11	F	59	21.0	59	C7	D	49	104	12.8	1.09	Yes
	**Mean** (***SD*)**	**N/A**	**50.5** **(10.7)**	**26.0** **(6.1)**	**78.5** **(93.0)**	**N/A**	**N/A**	**41.1** **(8.3)**	**126.8** **(20.5)**	**16.2** **(5.0)**	**0.64** **(0.51)**	**N/A**

*MST + tDCS_sham_, motor skill training plus sham transcranial direct current stimulation; MST+tDCS, motor skill training plus active transcranial direct current stimulation; M, male; F, female; BMI, body mass index (m/kg^2^); TSI, time since injury (months at enrollment); NLI, neurological level of injury; AIS, American Spinal Injury Association Impairment Scale classification; LEMS, lower extremity motor score (combined limbs); GXT HR_peak_, graded exercise test peak heart rate (bpm); GXT VO_2peak_, graded exercise test peak oxygen uptake (ml/kg/min).*

### Outcomes

#### Walking Measures

##### Overground Walking Speed

Analyses revealed a significant effect of TIME on walking speed, *F*(7, 161) = 11.69, *p* < 0.001. Neither GROUP nor TIME × GROUP interaction contributed to differences. In the absence of between-groups or interaction effects, *post hoc* comparisons for the main effect of TIME were performed for the full study sample. *Post hoc* comparisons revealed a significant increase in walking speed that persisted from D1 (*M* = 0.69 m/s, *SD* = 0.51) to D5 (*M* = 0.82 m/s, *SD* = 0.51), *t*(24) = 4.98, *p* < 0.001. A significant increase in walking speed was also observed over the 3-day intervention period from D2_pre_ (*M* = 0.75 m/s, *SD* = 0.50) to D4_post_ (*M* = 0.81 m/s, *SD* = 0.50), *t*(24) = 3.05, *p* = 0.006. Responsiveness of walking speed to intervention over the 5-day study period was large (*ES* = 1.04).

**FIGURE 4:**
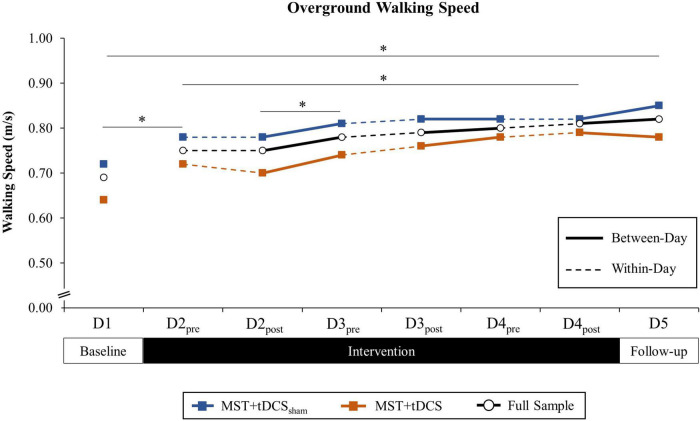
Overground walking speed (m/s) across all time points among the MST+tDCS_sham_ group (blue line with square marker), MST+tDCS group (orange line with square marker), and the combined study sample (black line with circle marker). Solid lines indicate between-day (offline), and hashed lines indicate within-day (online) time intervals during the intervention period. No between-groups differences were observed. *Significant difference between time points for the combined study sample (*p* < 0.10).

Paired comparisons of between-day time points revealed a significant increase in walking speed from D1 to D2_pre_ (*M* = 0.75 m/s, *SD* = 0.50), *t*(24) = 3.78, *p* = 0.001, and from D2_post_ (*M* = 0.75 m/s, *SD* = 0.50) to D3_pre_ (*M* = 0.78 m/s, *SD* = 0.48), *t*(24) = 2.36, *p* = 0.027. There were no differences in walking speed for within-day time points. Cumulative between-day change accounted for 84.6% of the overall change in walking speed from D1 to D5 (ΣΔM = 0.11 m/s, *SD* = 0.19), while within-day change accounted for 15.4% of the total change (ΣΔM = 0.02 m/s, *SD* = 0.14). Differences in cumulative within-day and between-day change were not statistically significant. Group-level paired comparisons of walking speed for the MST+tDCS_sham_ and MST+tDCS groups, along with the full study sample, are presented in Figure 4. Individual participant data for walking speed at each time point are provided in Supplementary Figure 1. Within-day and between-day change in walking speed is reported in Table 2.

**TABLE 2:** Between-day (blue columns), within-day (white columns), and cumulative (orange column) change in outcomes across time intervals.

	Time intervals							
	Baseline	Intervention					24-h follow-up	Cumulative
				
Outcomes	ΔD1-D2_pre_	ΔD2_pre_-D2_post_	ΔD2_post_-D3_pre_	ΔD3_pre_-D3_post_	ΔD3_post_-D4_pre_	ΔD4_pre_-D4_post_	ΔD4_post_-D5	ΔD1-D5
Walking speed (m/s)	0.06* **(0.08)** ***p* = 0.001**	0.00 (0.07) *p* = 0.740	0.03* **(0.07)** ***p* = 0.027**	0.02 (0.05) *p* = 0.160	0.01 (0.08) *p* = 0.728	0.01 (0.07) *p* = 0.502	0.01 (0.09) *p* = 0.704	0.13* **(0.13)** ***p* < 0.001**
Cadence (strides/min)	4.2* **(7.7)** ***p* = 0.012**	0.5 (4.3) *p* = 0.605	2.0* **(4.6)** ***p* = 0.039**	0.8 (3.8) *p* = 0.295	0.9 (5.8) *p* = 0.484	0.3 (5.1) *p* = 0.687	0.6 (5.6) *p* = 0.600	9.3* **(9.1)** ***p* < 0.001**
SL-weaker (cm)	5.7* **(7.1)** ***p* < 0.001**	−1.2 (6.5) *p* = 0.361	3.0* **(5.8)** ***p* = 0.017**	1.2 (5.2) *p* = 0.251	−0.4 (5.2) *p* = 0.708	0.4 (4.5) *p* = 0.552	0.0 (5.2) *p* = 0.899	8.7* **(9.1)** ***p* < 0.001**
SL-stronger (cm)	5.8* **(7.3)** ***p* < 0.001**	−0.7 (6.4) *p* = 0.579	2.6* **(5.7)** ***p* = 0.034**	1.4 (4.7) *p* = 0.133	−0.7 (5.2) *p* = 0.516	0.3 (4.9) *p* = 0.723	−0.2 (6.3) *p* = 0.797	8.3* **(9.0)** ***p* < 0.001**
TLA-weaker (°)	0.75* **(2.1)** ***p* = 0.081**	−0.22 (2.9) *p* = 0.716	0.57 (2.5) *p* = 0.259	−0.12 (1.8) *p* = 0.738	0.13 (1.8) *p* = 0.723	0.16 (1.4) *p* = 0.580	−0.61* **(1.5)** ***p* = 0.052**	0.67 (2.8) *p* = 0.237
TLA-stronger (°)	0.95 (2.8) *p* = 0.104	−0.18 (1.8) *p* = 0.619	0.10 (1.9) *p* = 0.787	0.86* **(1.9)** ***p* = 0.031**	−0.80* **(1.7)** ***p* = 0.026**	0.48 (1.5) *p* = 0.129	−0.07 (1.6) *p* = 0.829	1.34* **(2.8)** ***p* = 0.023**
ACC-weaker	0.02* **(0.00 to 0.05)** ***p* = 0.003**	0.00 (−0.03 to 0.01) *p* = 0.129	0.01* **(−0.01 to 0.03)** ***p* = 0.024**	0.00 (0.00 to 0.02) *p* = 0.378	−0.01 (−0.02 to 0.03) *p* = 0.125	0.00 (−0.01 to 0.02) *p* = 0.551	0.00 (−0.01 to 0.01) *p* = 0.843	0.01* **(0.00 to 0.43)** ***p* = 0.011**
ACC-stronger	0.01 (−0.01 to 0.01) *p* = 0.278	0.00 (−0.01 to 0.02) *p* = 0.537	0.01* **(0.00 to 0.02)** ***p* = 0.045**	0.00 (−0.01 to 0.02) *p* = 0.249	−0.01* **(−0.02 to 0.01)** ***p* = 0.037**	0.01 (−0.02 to 0.02) *p* = 0.586	−0.01 (−0.02 to 0.01) *p* = 0.180	0.01 (−0.02 to 0.01) *p* = 0.637
BBS (total score)								2.0* **(3.7)** ***p* = 0.010**
FES-I (total score)								−2.6* **(3.8)** ***p* = 0.002**

ACC reported as median (IQR). All other outcomes reported as mean (SD). *Significant difference between time points with corresponding data in bold (p values for walking speed, cadence, stride length, TLA, BBS, and FES-I derived from paired-samples t-test; p values for ACC derived from Wilcoxon signed-rank test). SL-Weaker, weaker limb stride length; SL-Stronger, stronger limb stride length; TLA-Weaker, weaker limb trailing limb angle; TLA-Stronger, stronger limb trailing limb angle; ACC-Weaker, weaker limb angular component of the coefficient of correspondence; ACC-Stronger, stronger limb angular component of the coefficient of correspondence; BBS, Berg Balance Scale; FES-I, Falls-Efficacy Scale-International version.

##### Cadence and Stride Length

Analyses revealed a significant effect of TIME on cadence, *F*(7, 160) = 12.71, *p* < 0.001, stronger limb stride length, *F*(7, 160) = 10.31, *p* < 0.001, and weaker limb stride length, *F*(7, 160) = 9.73, *p* < 0.001. There were no effects of GROUP or TIME × GROUP interaction on cadence or stride length of the stronger or weaker limb. Failing to observe between-groups differences or interaction effects, *post hoc* analyses were performed using data from the full study sample.

*Post hoc* analyses revealed a significant increase in cadence that persisted from D1 (*M* = 72.5 strides/min, *SD* = 35.5) to D5 (*M* = 81.8 strides/min, *SD* = 34.2), *t*(24) = 5.13, *p* < 0.001. Analyses of the 3-day intervention period revealed a significant increase in cadence from D2_pre_ (*M* = 76.7 strides/min, *SD* = 33.8) to immediately following intervention at D4_post_ (*M* = 81.2 strides/min, *SD* = 34.6), *t*(24) = 3.19, *p* = 0.004. Paired comparisons of between-day time points revealed significant increases in cadence from D1 to D2_pre_, *t*(24) = 2.70, *p* = 0.012, and from D2_post_ (*M* = 77.1 strides/min, *SD* = 34.0) to D3_pre_ (*M* = 79.1 strides/min, *SD* = 33.2), *t*(24) = 2.19, *p* = 0.039. There were no within-day differences in cadence. Differences in cumulative within-day and between-day change were not significant.

Significant increases were observed for stronger limb stride length from D1 (*M* = 101.3 cm, *SD* = 32.9) to D5 (*M* = 109.8 cm, *SD* = 32.2), *t*(24) = 4.58, *p* < 0.001, and for weaker limb stride length from D1 (*M* = 101.5 cm, *SD* = 33.3) to D5 (*M* = 110.2 cm, *SD* = 32.2), *t*(24) = 4.79, *p* < 0.001. Analyses of the 3-day intervention period revealed a significant increase in stronger stride length from D2_pre_ (*M* = 107.1 cm, *SD* = 32.1) to immediately following intervention at D4_post_ (*M* = 110.1 cm, *SD* = 31.4), *t*(24) = 2.06, *p* = 0.051, as well as weaker stride length from D2_pre_ (*M* = 107.2 cm, *SD* = 32.1) to D4_post_ (*M* = 110.4 cm, *SD* = 31.7), *t*(24) = 2.05, *p* = 0.051. Pairwise comparisons of between-day time points for stronger limb stride length revealed significant increases from D1 to D2_pre_, *t*(24) = 4.00, *p* = 0.001, and from D2_post_ (*M* = 106.4 cm, *SD* = 31.7) to D3_pre_ (*M* = 109.0 cm, *SD* = 30.7), *t*(24) = 2.25, *p* = 0.034. Likewise, between-day increases in weaker limb stride length were observed from D1 to D2_pre_, *t*(24) = 4.01, *p* = 0.001, and from D2_post_ (*M* = 106.0 cm, *SD* = 31.4) to D3_pre_ (*M* = 109.0 cm, *SD* = 31.1), *t*(24) = 2.57, *p* = 0.017. There were no within-day differences in stride length for the stronger or weaker limbs. Furthermore, differences in cumulative within-day and between-day change were not significant. Paired comparisons of cadence and stride length for the full study sample are reported in Table 2.

##### Trailing Limb Angle and ACC

Analyses revealed a significant effect of TIME, *F*(7, 161) = 3.10, *p* < 0.01, but no effect of GROUP or TIME × GROUP interaction on stronger limb TLA. There was no effect of TIME, GROUP, or TIME × GROUP interaction on weaker limb TLA. Analyses revealed a significant effect of TIME on stronger limb ACC, χ^2^(7) = 24.22, *p* = 0.001, and weaker limb ACC, χ^2^(7) = 23.40, *p* = 0.001. There were no GROUP differences in change in stronger or weaker limb ACC across time points. In the absence of GROUP differences, *post hoc* analyses for stronger limb TLA and weaker and stronger limb ACC were carried out using the full study sample.

*Post hoc* analyses revealed a significant increase in stronger limb TLA that persisted from D1 (*M* = 19.2°, *SD* = 4.5) to D5 (*M* = 20.5°, *SD* = 3.9), *t*(24) = 2.42, *p* = 0.023. There were no differences in stronger TLA from D2_pre_ to immediately following intervention at D4_post_. Pairwise comparisons of between-day time points revealed a significant decrease in stronger limb TLA from D3_post_ to D4_pre_ (*M* = 20.2°, *SD* = 3.9), *t*(24) = −2.25, *p* = 0.034, while within-day comparisons revealed a significant increase from D3_pre_ (*M* = 20.1°, *SD* = 4.5) to D3_post_ (*M* = 20.9°, *SD* = 3.4), *t*(24) = 2.29, *p* = 0.031.

**FIGURE 5:**
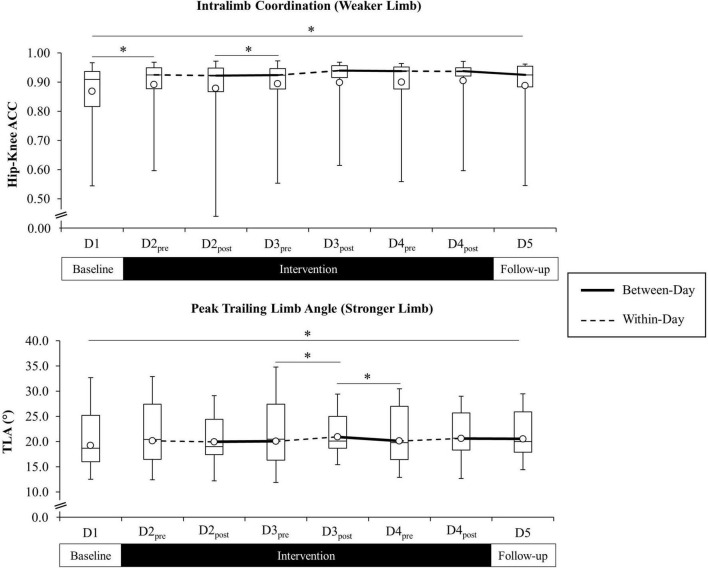
Weaker limb intralimb coordination (ACC) (**top** figure) and stronger limb trailing limb angle (TLA) (**bottom** figure) across all time points for the combined study sample. Open circle markers represent the mean at each time point. Solid lines indicate between-day (offline), and hashed lines indicate within-day (online) time intervals during the intervention period (line for the ACC reflected at the median). *Significant difference between time points (*p* < 0.10). Higher ACC values indicate improved cycle-to-cycle intralimb coordination (ACC = 1.0 indicates perfect cycle-to-cycle consistency in hip-knee relative motion).

*Post hoc* analyses revealed a significant increase in weaker limb ACC that persisted from D1 (Md = 0.91, IQR = 0.82–0.94) to D5 (Md = 0.93, IQR = 0.88–0.96), *Z* = −2.53, *p* = 0.011. Differences in weaker ACC were not significant from D2_pre_ to D4_post_. There were no differences in stronger limb ACC from D1 to D5 or from D2_pre_ to D4_post_. Pairwise comparisons of between-day time points revealed a significant increase in weaker limb ACC from D1 to D2_pre_ (Md = 0.93, IQR = 0.87–0.95), *Z* = −2.93, *p* = 0.003, and from D2_post_ (Md = 0.92, IQR = 0.87–0.95) to D3_pre_ (Md = 0.92, IQR = 0.88–0.96), *Z* = –2.26, *p* = 0.024. There were no within-day differences in weaker limb ACC. Pairwise comparison of between-day time points revealed a significant increase in stronger limb ACC from D2_post_ (Md = 0.93, IQR = 0.89–0.95) to D3_pre_ (Md = 0.94, IQR = 0.91–0.95), *Z* = −2.01, *p* = 0.045, and a decrease from D3_post_ (Md = 0.94, IQR = 0.92–0.96) to D4_pre_ (Md = 0.93, IQR = 0.91–0.95), *Z* = −2.08, *p* = 0.037. There were no within-day differences in stronger limb ACC. Paired comparisons of TLA (stronger limb) and ACC (weaker limb) for the full study sample are presented in Figure 5 and Table 2.

#### Balance Function

**FIGURE 6:**
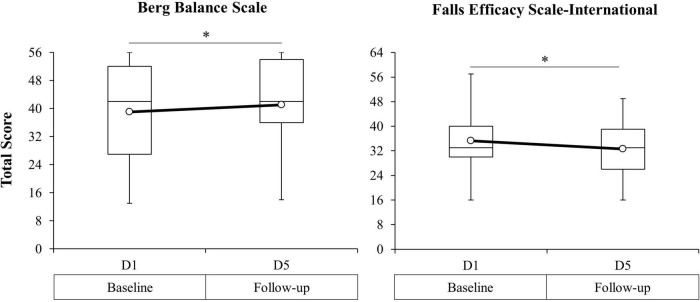
Berg Balance Scale (BBS) total score (**left** figure) and Falls Efficacy Scale-International (FES-I) total score (**right** figure) from baseline (D1) to 24-h post-intervention (D5) for the combined study sample. Open circle markers represent the mean at each time point. *Significant difference between time points (*p* < 0.10). Higher BBS scores indicate improved balance function. Lower FES-I scores indicate decreased self-reported fear of falling.

Analyses revealed a significant effect of TIME on the BBS, *F*(1, 23) = 7.16, *p* = 0.01, and FES-I, *F*(1, 23) = 12.43, *p* < 0.01. There was no effect of GROUP or TIME × GROUP interaction for either measure. In the absence of between-groups differences or interaction effects, *post hoc* analyses for the BBS and FES-I were carried out for the full study sample. Pairwise comparisons revealed a significant increase in BBS total score from D1 (*M* = 39.0, *SD* = 14.2) to D5 (*M* = 41.1, *SD* = 13.3), *t*(24) = 2.78, *p* = 0.01, and a decrease in FES-I total score from D1 (*M* = 35.3, *SD* = 10.2) to D5 (*M* = 32.6, *SD* = 8.8), *t*(24) = −3.52, *p* < 0.01. Paired comparisons of the BBS and FES-I for the full study sample are presented in Figure 6 and Table 2.

## Discussion

We examined the effects of a 3-day moderate-intensity MST program on measures of walking and balance function in participants with MISCI. Participation in brief MST was associated with significant increases in overground walking speed, cadence, bilateral stride length, stronger limb TLA, and weaker limb ACC. Measures of balance function and perceived fear of falling were also improved. We further examined whether combining MST with tDCS would lead to greater improvements in outcomes compared to MST alone. Concurrent application of tDCS failed to produce greater improvements in walking and balance function compared to MST plus sham tDCS. MST was associated with change in the primary outcome (walking speed) approaching or exceeding observations in longer-term locomotor training studies. Furthermore, among those walking outcomes that were positively influenced by MST intervention, between-day (offline) effects contributed to a greater percentage of total change in outcomes compared to within-day (online) effects.

### Improved Walking Performance Following Brief Motor Skill Training

Several randomized clinical trials involving various multi-week locomotor training interventions have reported improvements in overground walking speed ranging from 0.01 to 0.16 m/s (Alexeeva et al., 2011; Field-Fote and Roach, 2011; Jones et al., 2014b; Kapadia et al., 2014; Lotter et al., 2020). Despite the brief 3-day training period of our intensive MST program, our results were comparable to these prior studies (i.e., Δ walking speed = 0.13 m/s), although the magnitude of change did not reach the minimally clinically important difference of 0.15 m/s previously reported in this population (Forrest et al., 2014). Major limitations of prior investigations are the time and resources (e.g., capital, technology, personnel) needed to deploy the interventions. We investigated a motor skill training circuit of locomotor-related activities that required little to no equipment and could feasibly be implemented with supervision in the home or community. The MST program encouraged ballistic, cyclic volitional movement sequences that provoked a moderate-intensity cardiovascular response. These features of the MST circuit contrast with prevailing locomotor training approaches that emphasize guided massed practice stepping on a treadmill, either with or without bodyweight support or robotic assistance (Hannold et al., 2006; Field-Fote and Roach, 2011; Merholz et al., 2012). Aside from limitations on accessibility, these approaches often constrain the individual’s ability to actively explore motor solutions needed to successfully manage “real-world” environmental conditions (Mutha et al., 2011), such as the need to rapidly modulate step speed while traversing a crosswalk or modulating step height to successfully navigate a curb. While in our study the MST circuit was delivered in a clinical laboratory setting with a single person providing all supervision, the magnitude of improvement in overground walking speed over a short intervention period suggests value in further investigation. The MST intervention may be a viable supplement or alternative to locomotor training in cases where time and/or available resources are limited.

In addition to improvements in walking speed, participation in 3 days of MST was associated with significant increases in stride frequency (cadence) and length. Gait kinematics associated with walking impairments in persons with SCI have been reported extensively (Field-Fote and Tepavac, 2002; Pepin et al., 2003; Nooijen et al., 2009; Awai and Curt, 2014; Sohn et al., 2018; Ardestani et al., 2019). Both stride frequency and stride length are coupled to walking speed modulation in non-injured adults (van Hedel et al., 2006). In persons with SCI, muscle weakness and diminished capacity to produce high-velocity muscle actions of the lower limbs limit maximal walking speed, primarily through an effect on reduced cadence (Pepin et al., 2003). In a 12-week stratified, randomized study comparing four different overground and treadmill-based locomotor training strategies, both cadence and bilateral stride length were increased, regardless of approach (Nooijen et al., 2009). We observed an increase in cadence of 9.3 strides/min following only 3 days of training, which was comparable to the most effective approach examined in the previous study (i.e., overground training; 10.0 strides/min) and superior to all other modes of training examined (i.e., treadmill-based training; 3.0–7.8 strides/min).

Motor training interventions that emphasize high-velocity, ballistic volitional actions, as opposed to repetitive, constant velocity movements, may provide a more robust stimulus for increasing stride frequency in persons with SCI. In a case series report of persons with MISCI, 12-weeks of lower extremity resistance and ballistic plyometric training was associated with an increase in bilateral stride length but not cadence (Gregory et al., 2007). Failure of ballistic training to increase cadence in the case series report could be attributed to higher average baseline walking speeds among their small cohort (*M* = 1.08 m/s) compared to our sample (*M* = 0.69 m/s) as prior evidence suggests that maximal cadence plateaus as walking speeds approach 1.0 m/s in this population (Pepin et al., 2003). Longer training studies are needed to determine whether there is a ceiling effect for cadence following prolonged exposure to our MST circuit.

In addition to cadence and stride length, modulation of walking speed, in particular the transition from slow to fast walking, is aided by the individual’s ability to alter propulsive forces during locomotion (Peterson et al., 2011). In aging adults, smaller propulsive forces and an increased emphasis on more proximal leg muscles for power generation are associated with diminished walking capacity (i.e., speed and distance; Browne and Franz, 2018). In persons with stroke (Peterson et al., 2010; Awad et al., 2015, 2020) and SCI (Peters et al., 2018), impaired central drive (Wirth et al., 2008b; Bravo-Esteban et al., 2015; Awad et al., 2020) and muscle weakness (DiPiro et al., 2015; Roelker et al., 2019) contribute to decreased magnitude and rate of lower limb force development, culminating in diminished propulsive impulse and slower walking speeds. Interventions that increase corticospinal drive and facilitate volitional ability to rapidly recruit muscles of locomotion may enhance propulsive force generation and improve the capacity to modulate walking speed. Trailing limb angle has been validated as a surrogate measure of propulsive force in healthy adults (Hsiao et al., 2015) and individuals post-stroke (Lewek and Sawicki, 2019). To our knowledge, we are the first to characterize the effects of motor training on TLA during overground walking in persons with MISCI.

Baseline peak TLA of the stronger and weaker limbs among our sample (*M* = 19.2°) were lower than previous reports in non-injured adults (*M* = 26.3°) (Miyazaki et al., 2019). Three days of ballistic, cyclic MST was associated with a significant increase in stronger but not weaker TLA. The magnitude of change in stronger TLA (ΔM = 1.3°) is comparable to a 12-week study in persons with stroke, where treadmill and overground locomotor training was associated with an increase in both paretic and non-paretic TLA of approximately 2° (Hsiao et al., 2016). Ballistic training involving rapid, explosive muscle contractions is associated with greater task-specific adaptations compared to low-velocity training (Tschopp et al., 2011; Cordner et al., 2021). Increased corticospinal drive (Schubert et al., 2008), earlier onset motor unit activation (Van Cutsem et al., 1998), increased type II muscle fiber recruitment (Wilson et al., 2012), and increased rate of force development (Del Vecchio et al., 2019) are aspects of ballistic training that may support improved forward propulsion kinematics and facilitate walking speed modulation in persons with motor deficits. These features of training may account for the comparable changes in TLA we observed despite the marked differences in training mode and duration; however, the effects of the MST circuit in persons with MISCI may be restricted to the less impaired limb, where greater corticospinal tract preservation may contribute to enhanced ability to modulate lower limb kinematics (Hope et al., 2020). Although evidence in non-injured adults suggests that even small-scale changes in TLA (i.e., Δ = 1.5°) can contribute to as much as 65% of the increase in propulsive force generated during slow to fast walking (Hsiao et al., 2015), it is unclear whether the modest increase in TLA we observed was consequential to increases in walking speed among our participants. Further analysis is needed to uncover potential relationships between changes in TLA and walking speed in this population.

Prior investigations have examined intralimb coordination (hip-knee ACC) as a measure of the integrated function of motor systems involved in cyclic locomotor behavior in persons with SCI (Field-Fote and Tepavac, 2002; Nooijen et al., 2009; Awai and Curt, 2014; Holleran et al., 2018). In a case series report, up to 40 sessions of treadmill and overground step training was associated with both positive and negative changes in weaker limb ACC (Δ range = −0.07 to 0.17; Holleran et al., 2018). We observed similar results among our larger sample following 3 days of MST (Δ range = −0.01 to 0.13), indicating a similar degree of variability in responsiveness among participants, regardless of training mode or duration. In a 12-week study of treadmill-based locomotor training combined with peroneal nerve stimulation, 64% of participants (9/14) demonstrated improved intralimb coordination of the weaker limb with an average increase in ACC of 0.09 (Field-Fote and Tepavac, 2002). Our findings were comparable in that 68% of participants (17/25) demonstrated improvements in weaker limb ACC, albeit the mean change among our sample was smaller (ΔM = 0.03). Finally, 36-sessions of high-intensity treadmill training was associated with a non-significant increase in weaker limb ACC (ΔM = 0.04) (Leech et al., 2016). We observed a significant increase in median weaker limb ACC (ΔMd = 0.01), with mean change being comparable to the latter study.

In light of the above, we draw three conclusions concerning intralimb coordination. First, response of weaker limb ACC to training was similar in terms of range and magnitude of change reported across studies, regardless of training approach. This suggests that adaptation of cycle-to-cycle intralimb coordination in persons with MISCI is not limited to participation in treadmill-based massed practice stepping protocols and that alternative motor training approaches may yield similar benefits. Second, although we observed a sample-wide increase in weaker limb ACC, the magnitude of change was small and not all participants demonstrated improvement. Moreover, we failed to observe a change in stronger limb ACC. It is likely that responsiveness to training is participant and limb dependent and may be contingent upon one or more individual characteristics, such as the degree of preserved lower limb motor function, presence and severity of spasticity, and/or baseline functional capacity. In fact, there was marked heterogeneity among participants both within and between studies in terms of baseline LEMS, walking speeds, and ACC, which could account for the variable responses. Finally, the consistent finding of limited and modest ranges of responses among the present and prior studies suggests that intralimb coordination may not be easily altered with motor training despite significant change in other measures of walking ability. For example, we observed marked increases in stride frequency and stride length that coincided with significant increases in overground walking speed, yet cycle-to-cycle intralimb coordination was little changed. This could indicate that the ongoing coordination, relative timing, and maintenance of lower limb segment positions is generally conserved, even in the face of significant changes in other kinematic variables important for walking speed modulation.

### Improved Balance and Reduced Fear of Falling Following Brief Motor Skill Training

Participation in three consecutive days of MST was associated with significant improvements in balance function (increased BBS scores) and reduced fear of falling (decreased FES-I scores). Muscle weakness, impaired balance, and concern for falling contribute to a higher frequency of falls among persons with SCI who are ambulatory (Jorgensen et al., 2017; Kahn et al., 2019). Enhanced balance performance is associated with improved overground walking ability, reduced incidence of falls, and enhanced confidence in performing activities of daily living (Datta et al., 2009; Forrest et al., 2012; Singh et al., 2021). The finding that brief MST was associated with a significant improvement in balance is consistent with longer-term training interventions in participants with MISCI (Stevens et al., 2015; Morrison et al., 2018; Neville et al., 2019; Houston et al., 2020), although the magnitude of change we observed (ΔBBS = 2.0) was smaller than these previous reports (ΔBBS range = 4.5–13.0). Differences in the magnitude of change between studies may be due to heterogeneity in baseline motor function, time since injury at intervention onset, and total training duration. An important distinction between the present and prior interventions is the time, equipment, and personnel needed to realize improvements in balance performance. A longer-term study, including individuals with greater balance impairments, is needed to determine whether the magnitude of effect of the MST intervention on upright balance would be comparable to more traditional motor training approaches.

Despite existing evidence linking fear of falling with diminished functional walking capacity (Gabner et al., 2021), impaired balance (Wirz et al., 2010), and increased falls risk (Jorgensen et al., 2017), the effect of motor training on fear of falling has not been extensively characterized in persons with SCI (Abou et al., 2021). As a result, it is not clear how much improvement in the FES-I could be expected with training or to what extent changes in this measure are related to meaningful improvements in functional outcomes in this population (e.g., walking and balance performance). A single randomized controlled trial involving 6-weeks of thrice-weekly unsupported seated balance training was associated with a 2-point reduction in the FES-I (Boswell-Ruys et al., 2010); however, the effect of intervention was not different from a non-exercise control group. We observed a similar reduction in fear of falling (ΔFES-I = −2.6) that coincided with improvements in walking and balance function following 3 days of upright motor training, suggesting a possible relationship between the perception of falling and upright functional capacity. Further investigation into the potential interaction between these outcomes following longer-term intervention would be of value.

### Transcranial Direct Current Stimulation Did Not Augment Effects of Lower Extremity Motor Training

Despite significant differences in walking and balance outcomes following brief MST, concurrent application of tDCS failed to augment the effects of training. Although existing evidence indicates that anodal tDCS applied to the motor cortex enhances corticospinal excitability in healthy adults and in persons with stroke (Bastani and Jaberzadeh, 2012), and that this effect supports mechanisms that promote motor skill acquisition and consolidation (Wittkopf et al., 2021), recent evidence suggests a high degree of interindividual variability in motor cortical responsiveness to tDCS (Jonker et al., 2021), which may have contributed to lack of effect in the present study. Furthermore, a recent meta-analysis examining effectiveness of active- vs. sham tDCS on improving motor function after incomplete SCI revealed a significant but small effect in favor of active-tDCS (de Araujo et al., 2020); however, only five studies were included in the analysis and only two studies combined tDCS with lower extremity motor training (Kumru et al., 2016; Raithatha et al., 2016). Both studies applied tDCS in combination with robotic-assisted treadmill training, and while improvements in walking ability were observed in both studies, neither reported between-groups differences in outcomes. Our findings are consistent with these previous reports as equivalent improvements in walking ability were observed in both the MST+tDCS and MST+tDCS_sham_ groups.

There may be several reasons for our failure to observe effects of tDCS. First, damage to spinal tracts is highly heterogenous even among persons with similar AIS classification; as such, differences in extent of corticospinal tract damage among participants may have influenced individual responsiveness to tDCS. The increased corticomotor excitability associated with anodal tDCS is believed to augment volitional motor control by increasing descending drive (Nitsche and Paulus, 2000). The extent of damage to descending tracts may have limited transmission between cortical and spinal circuits in our sample. Future studies may be improved by use of motor-evoked potentials as a probe of responsiveness to corticomotor stimulation (Labruna et al., 2019) and to stratify individuals according to responders and non-responders based on the magnitude of these evoked responses. Second, our measures of motor function (i.e., walking and balance outcomes) may not have been sufficiently sensitive to capture more discrete changes in volitional motor actions previously reported to be influenced by acute changes in corticospinal excitability. For example, the motor cortex drives the ankle dorsiflexors on a step-by-step basis during bipedal walking (Peterson et al., 2012), and a single session of tDCS applied to the motor cortex is associated with enhanced ankle control in participants with MISCI (Yamaguchi et al., 2016). In the present study, if tDCS did influence ankle motor control, the effect did not manifest as improved responses in walking and balance performance among participants who received active tDCS. Based on our findings, combining MST with tDCS did not confer greater advantage on lower extremity motor tasks involving upright mobility compared to MST alone.

### Within-Day (Online) vs. Between-Day (Offline) Effects

It is well-documented that training-induced procedural learning is achieved through a combination of online [acquisition (within-day)] and offline [consolidation (between-day)] processes (Robertson et al., 2004; Dayan and Cohen, 2011). This study is the first to characterize within-day and between-day changes in walking outcomes over multiple consecutive days of lower extremity motor training in persons with MISCI, and to assess the effect of adding tDCS. Offline effects of intensive MST accounted for the largest percentage of total change in walking speed, cadence, and stride length. In addition, change in these measures was only observed during offline time intervals. These effects were not further influenced by the addition of tDCS.

Aerobic exercise and non-invasive brain stimulation have each been explored as potential neuromodulation approaches that have the capacity to reinforce mechanisms that subserve neuroplasticity and promote motor learning (Nitsche et al., 2003; Singh et al., 2014b; Skriver et al., 2014; Holman and Staines, 2021; Kim et al., 2021a). Intensive aerobic exercise facilitates neuroplasticity through various mechanisms, including enhanced blood flow to the motor cortex (Singh and Staines, 2015), upregulation of glucocorticoids (Milani et al., 2010) and neurotrophic factors (He et al., 2013), and increased states of arousal (McMorris et al., 2015). A single-session of moderate- to high-intensity aerobic exercise is associated with an increase in brain-derived neurotrophic factor (Mang et al., 2014), enhanced long-term potentiation-like plasticity (Singh et al., 2014b), and greater retention of skilled motor tasks (Holman and Staines, 2021). These processes are believed to augment motor skill training through an effect on motor program consolidation (offline processes) (Wanner et al., 2020). Likewise, anodal tDCS applied to the motor cortex induces changes in neuronal excitability that outlast the stimulation period (Monte-Silva et al., 2013), and when combined with motor training, is believed to facilitate learning through NMDA-dependent long-term potentiation and an effect on motor skill consolidation (Cocco et al., 2018).

While intensive MST was associated with significant improvements in measures of walking performance that appeared to be reinforced during the 24-h interval between training days, we failed to observe an additive effect of tDCS on either online or offline motor performance. The finding concerning lack of effect of tDCS on motor performance consolidation contrasts with prior motor training studies in non-injured adults. For example, both single session (Kim et al., 2021b) and multi-day (Reis et al., 2009) application of tDCS combined with upper limb motor training is associated with greater offline, but not online, improvements in motor performance when compared to sham tDCS. Likewise, in the lower limb, tDCS combined with motor training is associated with greater post-intervention (offline) improvements in a visuomotor stepping activity (Tseng et al., 2020) and a visuomotor tracking task (Sriraman et al., 2014) compared to sham stimulation. Despite these studies, there is a paucity of research investigating the temporal influence of tDCS on functional motor performance in persons with SCI, but our findings suggest that previous observations of offline consolidation effects of tDCS may not hold true for walking-related outcomes in this population.

It should be noted that much, but not all, of the offline change in walking outcomes following MST occurred between baseline (D1) and immediately prior to the first day of intervention on Day-2 (D2_pre_). We propose two possible explanations to account for this observation. First, it is possible that the inclusion of a maximal aerobic exercise test at D1 facilitated processes that enhanced learning of the walking test at D2_pre_. At baseline, participants completed multiple bouts of fast walking, followed by upright standing balance tests, and ending with an intensive maximal graded exercise test to fatigue. Evidence highlighting the influence of intensive exercise on mechanisms that promote motor learning has been detailed in a previous section. It is possible that baseline aerobic exercise testing induced physiological mechanisms that augmented improvements in walking performance at D2_pre_. It may be prudent in future studies to conduct aerobic exercise testing on a separate day from outcomes testing to avoid possible influence of acute exercise on subsequent tests of motor performance. Second, we cannot rule out presence of a simple learning effect wherein performing the walking test at D1 resulted in improved performance at D2_pre_. Future studies may be improved by including multiple baseline assessments at different time points to establish stable pre-training levels of walking performance prior to intervention.

## Limitations

Despite being the largest available study of intensive, high-velocity, volitional lower extremity motor training to improve walking outcomes in persons with SCI, and the largest study of tDCS to augment lower extremity function in this population, several limitations are considered. First, due to COVID-19 restrictions, our recruitment goal of 30 participants was not reached (*n* = 25) and between-groups sample sizes were unbalanced, which may have contributed to an inability to detect between-groups differences; however, statistical modeling has indicated that, in pilot studies, a sample size of 12 participants per group is sufficient for estimating mean change and variability in outcomes (Julious, 2005). Even if statistically significant between-groups differences were detected in the present study, it is doubtful that the magnitude of these differences would have been clinically meaningful. Second, mean baseline walking speed and total LEMS differed between groups, favoring the MST+tDCS_*sham*_ group in the former case and the MST+tDCS group in the latter. Furthermore, these measures were higher among our participants compared to some prior studies. Existing evidence from persons with SCI indicates that lower extremity strength is associated with walking speed (DiPiro et al., 2015), and baseline walking speed may be a predictor of responsiveness to training (Jones et al., 2014a). Third, various tDCS electrode montages have been proposed for modulating underlying neurophysiology, although the optimal montage has yet to be determined. The findings should be viewed within the context of the specific montage we used. Furthermore, it has been reported that sham tDCS may exert a small effect on corticospinal excitability (Dissanayaka et al., 2018); however, there is little evidence that transient shifts in neuronal excitability induced by sham stimulation are sufficient to produce changes in functional outcomes. Finally, the absence of a non-MST control group complicates interpretation of the unique contributions of the MST intervention. The addition of such a group could strengthen future studies.

## Conclusion

Restoration of motor function, including walking and balance, is central to rehabilitation for persons with MISCI and has implications for long-term health and independence for persons with motor deficits resulting from spinal injury. The time, effort, and costs associated with obtaining meaningful improvements in function after injury are of ongoing interest. The MST circuit was designed to capitalize on the physiologic evidence for intensive training to activate neuroplastic mechanisms and to increase corticospinal drive through volitional engagement. Furthermore, the intent was to address limitations posed by traditional locomotor training approaches that emphasize slow, repetitive massed-practice stepping, lengthy training periods, and costly equipment that lacks long-term accessibility. While no effect of tDCS was identified, the cyclic, ballistic training activities appear to promote improvements in walking speed, relevant gait kinematics, and balance performance. Larger studies with longer training periods are needed to determine whether greater improvements in function can be realized.

## Data Availability Statement

The raw data supporting the conclusions of this article will be made available by the authors, without undue reservation.

## Ethics Statement

The study protocol was approved by the Institutional Review Board at Shepherd Center in accordance with the Declaration of Helsinki. Individuals enrolled in the study provided written informed consent prior to participation.

## Author Contributions

EF-F conceptualized the study and its design. NE and CS performed data collection and statistical analyses. EF-F and NE drafted the manuscript. EF-F, NE, and CS contributed to revisions and approved the final version for submission. All authors contributed to the article and approved the submitted version.

## Conflict of Interest

The authors declare that the research was conducted in the absence of any commercial or financial relationships that could be construed as a potential conflict of interest.

## Publisher’s Note

All claims expressed in this article are solely those of the authors and do not necessarily represent those of their affiliated organizations, or those of the publisher, the editors and the reviewers. Any product that may be evaluated in this article, or claim that may be made by its manufacturer, is not guaranteed or endorsed by the publisher.

## Abbreviations

10MWT: 10-meter walk test
ACC: angular component of the coefficient of correspondence
BBS: Berg Balance Scale
BDNF: brain-derived neurotrophic factor
D1: day-1 baseline test
D2_pre_: day-2 pre-test
D2_post_: day-2 post-test
D3_pre_: day-3 pre-test
D3_post_: day-3 post-test
D4_pre_: day-4 pre-test
D4_post_: day-4 post-test
D5: day-5 24-h post-intervention test
FES-I: Falls Efficacy Scale-International.
GXT: graded exercise test
HR_avg_: average heart rate
HR_peak_: peak heart rate
HRR: heart rate reserve
LEMS: lower extremity motor score
MISCI: motor-incomplete spinal cord injury
MST: motor skill training
MST+tDCS_sham_: motor skill training plus sham tDCS stimulation
MST+tDCS: motor skill training plus active transcranial direct current stimulation
SCI: spinal cord injury
tDCS: transcranial direct current stimulation
TLA: trailing limb angle.

## References

[B1] Abou L. Alluri A. Fliflet A. Du Y. Rice L. (2021). Effectiveness of physical therapy interventions in reducing fear of falling among individuals with neurologic diseases: a systematic review and meta-analysis. *Arch. Phys. Med. Rehabil.* 102 132–154. 10.1016/j.apmr.2020.06.025 32745544

[B2] ACSM Riebe D. Ehrman J. Ligouri G. Magal M. (2018). *ACSM’s Guidelines For Exercise Testing And Prescription.* Philadelphia, PA: Wolters Kluwer.

[B3] Alexeeva N. Sames C. Jacobs P. Hobday L. DiStasio M. Mitchell S. (2011). Comparison of training methods to improve walking in persons with chronic spinal cord injury: a randomized clinical trial. *J. Spinal Cord Med.* 34 362–379. 10.1179/2045772311Y.0000000018 21903010 PMC3152808

[B4] Ardestani M. Henderson C. Salehi A. Mahtani G. Schmit B. Hornby T. (2019). Kinematic and neuromuscular adaptations in incomplete spinal cor dinjury after high- versus low-intensity locomotor training. *J. Neurotrauma* 36 2036–2044. 10.1089/neu.2018.5900 30362878 PMC6599383

[B5] Awad L. Binder-Macleod S. Pohlig R. Reisman D. (2015). Paretic propulsion and trailing limb angle are key determinants of long-distance walking function after stroke. *Neurorehabil. Neural Repair* 29 499–508. 10.1177/1545968314554625 25385764 PMC4426250

[B6] Awad L. Hsiao H. Binder-Macleod S. (2020). Central drive to the paretic ankle plantarflexors affects the relationship between propulsion and walking speed after stroke. *J. Neurol. Phys. Ther.* 44 42–48. 10.1097/NPT.0000000000000299 31834220 PMC8049399

[B7] Awai L. Curt A. (2014). Intralimb coordination as a sensitive indicator of motor-control impairment after spinal cord injury. *Front. Hum. Neurosci.* 8:148. 10.3389/fnhum.2014.00148 24672464 PMC3956041

[B8] Bastani A. Jaberzadeh S. (2012). Does anodal transcranial direct current stimulation enhance exctability of the motor cortex and motor function in healthy individuals and subjects with stroke: a systematic review and meta-analysis. *Clin. Neurophysiol.* 123 644–657. 10.1016/j.clinph.2011.08.029 21978654

[B9] Boswell-Ruys C. Harvey L. Barker J. Ben M. Middleton J. Lord S. (2010). Training unsupported sitting in people with chronic spinal cord injuries: a randomized controlled trial. *Spinal Cord* 48 138–143. 10.1038/sc.2009.88 19597520

[B10] Bravo-Esteban E. Taylor J. Avila-Martin G. Simon-Martinez C. Torricelli D. Pons J. (2015). “Tibilais anterior electromyographic anlaysis during fast dorsiflexion: relationship with recovery of gait, muscle strength and evoked potentials during subacute spinal cord injury,” in *Proceedings of the 2015 7th International IEEE/EMBS Conference on Neural Engineering (NER)*, Montpellier.

[B11] Browne M. Franz J. (2018). More push from your push-off: joint-level modifications to modulate propulsive forces in old age. *PLoS One* 13:e0201407. 10.1371/journal.pone.0201407 30089143 PMC6082565

[B12] Celnik P. (2015). Understanding and modulating motor learning with cerebellar stimulation. *Cerebellum* 14 171–174. 10.1007/s12311-014-0607-y 25283180 PMC4348328

[B13] Cobianchi S. Arbat-Plana A. Lopez-Alvarez V. Navarro X. (2017). Neuroprotective effects of exercise treatments after injury: the dual role of neurotrophic factors. *Curr. Neuropharmacol.* 15 495–518. 10.2174/1570159X14666160330105132 27026050 PMC5543672

[B14] Cocco S. Podda M. Grassi C. (2018). Role of BDNF signaling in memory enhancement induced by transcranial direct current stimulation. *Front. Neurosci.* 12:427. 10.3389/fnins.2018.00427 29997473 PMC6028595

[B15] Cordner T. Egerton T. Schubert K. Wijesinghe T. Williams G. (2021). Ballistic resistance training: feasibility, safety, and effectiveness for improving mobility in adults with neurologic conditions: a systematic review. *Arch. Phys. Med. Rehabil.* 102 735–751. 10.1016/j.apmr.2020.06.023 32745546

[B16] Cortes M. Medeiros A. Gandhi A. Lee P. Krebs H. Thickbroom G. (2017). Improved grasp function with transcranial direct current stimulation in chronic spinal cord injury. *NeuroRehabilitation* 41 51–59. 10.3233/NRE-171456 28505987

[B17] Crozier K. Cheng L. Graziani V. Zorn G. Herbison G. Ditunno J. (1992). Spinal cord injury: prognosis for ambulation based on quadriceps recovery. *Paraplegia* 30 762–767. 10.1038/sc.1992.147 1484726

[B18] Datta S. Lorenz D. Morrison S. Ardolino E. Harkema S. (2009). A multivariate examination of temporal changes in Berg Balance Scale items for patients with ASIA impairment scale C and D spinal cord injuries. *Arch. Phys. Med. Rehabil.* 90 1208–1217. 10.1016/j.apmr.2008.09.577 19577035

[B19] Dayan E. Cohen L. (2011). Neuroplasticity subserving motor skill learning. *Neuron* 72 443–454. 10.1016/j.neuron.2011.10.008 22078504 PMC3217208

[B20] de Araujo A. Ribeiro F. Massetti T. Potter-Baker K. Cortes M. Plow E. (2020). Effectiveness of anodal transcranial direct current stimulation to improve muscle strength and motor functionality after incomplete spinal cord injury: a systematic review and meta-analysis. *Spinal Cord* 58 635–646. 10.1038/s41393-020-0438-2 32066873

[B21] Del Vecchio A. Negro F. Holobar A. Casolo A. Folland J. Felici F. (2019). You are as fast as your motor neruons: speed of recruitment and maximal discharge of motor neurons determine the maximal rate of force development. *J. Physiol.* 9 2445–2456. 10.1113/JP277396 30768687 PMC6487919

[B22] Dewan N. MacDermid J. (2014). Falls efficacy scale - international (FES-I). *J. Physiother.* 60:60. 10.1080/09593985.2021.1901327 24856947

[B23] Dinoff A. Herrmann N. Swardfager W. Liu C. Sherman C. Chan S. (2016). The effect of exercise training on resting concentrations of peripheral brain-derived neurotrophic factor (BDNF): a meta-analysis. *PLoS One* 11:e0163037. 10.1371/journal.pone.0163037 27658238 PMC5033477

[B24] DiPiro N. Holthaus K. Morgan P. Embry A. Perry L. Bowden M. (2015). Lower extremity strength is correlated with walking function after incomplete SCI. *Top. Spinal Cord Inj. Rehabil.* 21 133–139. 10.1310/sci2102-133 26364282 PMC4568094

[B25] Dissanayaka T. Zoghi M. Farrell M. Egab G. Jaberzadeh S. (2018). Sham transcranial electrical stimulation and its effect on corticospinal excitability: a systematic review and meta-analysis. *Rev. Neurosci.* 29 223–232. 10.1515/revneuro-2017-0026 28889119

[B26] El-Sayes J. Harasym D. Turco C. Locke M. Nelson A. (2019). Exercise-induced neuroplasticity: a mechanistic model and prospects for promoting plasticity. *Neuroscientist* 25 65–85. 10.1177/1073858418771538 29683026

[B27] Estes S. Iddings J. Field-Fote E. (2017). Priming neural circuits to modulate spinal reflex excitability. *Front. Neurol.* 8:17. 10.3389/fneur.2017.00017 28217104 PMC5289977

[B28] Evans N. Field-Fote E. (2022). A pilot study of intensive locomotor-related skill training and transcranial direct current stimulation in chronic spinal cord injury. *J. Neurol. Phys. Ther.* (in press). 10.1097/NPT.0000000000000403 35544283

[B29] Feise R. (2002). Do multiple outcome measures require p-value adjustment? *BMC Med. Res. Methodol.* 2:8. 10.1186/1471-2288-2-8 12069695 PMC117123

[B30] Field-Fote E. Roach K. (2011). Influence of a locomotor training approach on walking speed and distance in people with chronic spinal cord injury: a randomized clinical trial. *Phys. Ther.* 91 48–60. 10.2522/ptj.20090359 21051593 PMC3017322

[B31] Field-Fote E. Tepavac D. (2002). Improved intralimb coordination in people with incomplete spinal cord injury following training with body weight support and electrical stimulation. *Phys. Ther.* 82 707–715. 10.1093/ptj/82.7.707 12088467

[B32] Fisher B. Wu A. Salem G. Song J. Lin C. Yip J. (2008). The effect of exercise training in improving motor performance and corticomotor excitability in people with early Parkinson’s disease. *Arch. Phys. Med. Rehabil.* 89 1221–1229. 10.1016/j.apmr.2008.01.013 18534554 PMC2989816

[B33] Forrest G. Hutchinson K. Lorenz D. Buehner J. VanHiel L. Sisto S. (2014). Are the 10 meter and 6 minute walk tests redundant in patients with spinal cord injury? *PLoS One* 9:e94108. 10.1371/journal.pone.0094108 24788068 PMC4006773

[B34] Forrest G. Lorenz D. Hutchinson K. VanHiel L. Basso M. Datta S. (2012). Ambulation and balance outcomes measure different aspects of recovery in individuals with chronic, incomplete spinal cord injury. *Arch. Phys. Med. Rehabil.* 93 1553–1564. 10.1016/j.apmr.2011.08.051 22920452

[B35] Gabner H. List J. Martindale C. Regensburger M. Klucken J. Winkler J. (2021). Functional gait measures correlate with fear of falling, and quaility of life in patients with hereditary spastic paraplegia: a cross-sectional study. *Clin. Neurol. Neurosurg.* 209:106888. 10.1016/j.clineuro.2021.106888 34455170

[B36] Galea J. Jayaram G. Celnik P. (2009). Modulation of cerebellar excitability by polarity-specific nonivasive direct current stimulation. *J. Neurosci.* 29 9115–9122. 10.1523/JNEUROSCI.2184-09.2009 19605648 PMC2760225

[B37] Gasper R. Padula N. Freitas T. de Oliveira J. Torriani-Pasin C. (2019). Physical exercise for individuals with spinal cord injury: systematic review based on the international classification of functioning, disability, and health. *J. Sport Rehabil.* 28 505–516. 10.1123/jsr.2017-0185 30300056

[B38] Gomes-Osman J. Field-Fote E. (2015). Cortical vs. afferent stimulation as an adjuct to functional task practice training: a randomized, comparitive pilot study in people with cervical spinal cord injury. *Clin. Rehabil.* 29 771–782. 10.1177/0269215514556087 25381344

[B39] Gregory C. Bowden M. Jayaraman A. Shah P. Behrman A. Kautz S. (2007). Resistance training and locomotor recovery after incomplete spinal cord injury: a case series. *Spinal Cord* 45 522–530. 10.1038/sj.sc.3102002 17228358

[B40] Guo W. Ji Y. Wang S. Sun Y. Lu B. (2014). Neuronal activity alters BDNF-TrkB signaling kinetics and downstream functions. *J. Cell Sci.* 127 2249–2260. 10.1242/jcs.139964 24634513

[B41] Guo W. Nagappan G. Lu B. (2018). Differential effects of transient and sustained activation of BDNF-TrkB signaling. *Dev. Neurobiol.* 78 647–659. 10.1002/dneu.22592 29575722

[B42] Hannold E. Young M. Rittman M. Bowden M. Behrman A. (2006). Locomotor training: experiencing the changing body. *J. Rehabil. Res. Dev.* 43 905–916. 10.1682/jrrd.2005.07.0122 17436176

[B43] He Y. Zhang X. Yung W. Zhu J. Wang J. (2013). Role of BDNF in central motor structures and motor diseases. *Mol. Neurobiol.* 48 783–793. 10.1007/s12035-013-8466-y 23649659

[B44] Higgins S. (1991). Motor skill acquisition. *Physical Therapy* 71 123–139. 1989008 10.1093/ptj/71.2.123

[B45] Hiremath S. Hogaboom N. Raoscher M. Worobey L. Oyster M. Boninger M. (2017). Longitudinal prediction of quality-of-life scores and locomotion in indviduals with traumatic spinal cord injury. *Arch. Phys. Med. Rehabil.* 98 2385–2392. 10.1016/j.apmr.2017.05.020 28647550

[B46] Holleran C. Hennessy P. Leddy A. Mahtani G. Brazg G. Schmit B. (2018). High-intensity variable stepping training in patients with motor incomplete spinal cord injury: a case series. *J. Neurol. Phys. Ther.* 42 94–101. 10.1097/NPT.0000000000000217 29547484 PMC7128539

[B47] Holman S. Staines W. (2021). The effect of acute aerobic exercise on the consolidation of motor memories. *Exp. Brain Res.* 239 2461–2475. 10.1007/s00221-021-06148-y 34114077

[B48] Hope J. Koter R. Estes S. Field-Fote E. (2020). Disrupted ankle control and spasticity in persons with spinal cord injury: the association between neurophysiologic measures and function. A scoping review. *Front. Neurol.* 11:166. 10.3389/fneur.2020.00166 32218765 PMC7078326

[B49] Houston D. Lee J. Unger J. Masani K. Musselman K. (2020). Functional electrical stimualtion plus visual feedback balance training for standing balance performance among individuals with incomplete spinal cord injury: a case series. *Front. Neurol.* 11:680. 10.3389/fneur.2020.00680 32793101 PMC7390869

[B50] Hsiao H. Knarr B. Higginson J. Binder-Macleod S. (2015). The relative contribution of ankle moment and trailing limb angle to propulsive force during gait. *Hum. Mov. Sci.* 39 221–221. 10.1016/j.humov.2014.11.008 25498289 PMC4272868

[B51] Hsiao H. Knarr B. Pohlig R. Higginson J. Binder-Macleod S. (2016). Mechanisms used to increase peak propulsive force following 12-weeks of gait training in individuals poststroke. *J. Biomech.* 49 388–395. 10.1016/j.jbiomech.2015.12.040 26776931 PMC4761516

[B52] IBM Corp. (2020). *IBM SPSS Statistics for Windows, Version 27.0.* Armonk, NY: IBM Corp.

[B53] Inoue T. Ninuma S. Hayashi M. Okuda A. Asaka T. Maejima H. (2018). Effects of long-term exercise and low-level inhibition of GABAergic synapses on motor control and the expression of BDNF in the motor related cortex. *Neurol. Res.* 40 18–25. 10.1080/01616412.2017.1382801 29019708

[B54] Jakeman L. Wei P. Guan Z. Stokes B. (1998). Brain-derived neurotrophic factor stimulates hindlimbg stepping and sprouting of cholinergic fibers after spinal cord injury. *Exp. Neurol.* 154 170–184. 10.1006/exnr.1998.6924 9875278

[B55] John L. Cherian B. Babu A. (2010). Postural control and fear of faliing in perosns with low-level paraplegia. *J. Rehabil. Res Dev.* 47 497–502. 10.1682/jrrd.2009.09.0150 20803393

[B56] Jones M. Evans N. Tefertiller C. Backus D. Sweatman M. Tansey K. (2014b). Activity-based therapy for recovery of walking in individuals with chronic spinal cord injury: results from a randomized clinical trial. *Arch. Phys. Med. Rehabil.* 95 2239–2246. 10.1016/j.apmr.2014.07.400 25102384

[B57] Jones M. Evans N. Tefertiller C. Backus D. Sweatman M. Tansey K. (2014a). Activity-based therapy for recovery of walking in chronic spinal cord injury: results from a secondary analysis to determine responsiveness to therapy. *Arch. Phys. Med. Rehabil.* 95 2247–2252. 10.1016/j.apmr.2014.07.401 25102385

[B58] Jonker Z. Gaiser C. Tulen J. Ribbers G. Frens M. Selles R. (2021). No effect of anodal tDCS on motor cortical excitability and no evidence for repsonders in a large double-blind placebo-controlled trial. *Brain Stimul.* 14 100–109. 10.1016/j.brs.2020.11.005 33197654

[B59] Jorgensen V. Forslund E. Opheim A. Franzen E. Wahman K. Hultling C. (2017). Falls and fear of falling predict future falls and related injuries in ambulatory people with spinal cord injury: a longitudinal observational study. *J. Physiother.* 63 108–113. 10.1016/j.jphys.2016.11.010 28343914

[B60] Julious S. (2005). Sample size of 12 per group rule of thumb for a pilot study. *Pharmac. Statist.* 4 287–291. 10.1002/pst.185

[B61] Kahn A. Pujol C. Laylor M. Unic N. Pakosh M. Dawe J. (2019). Falls after spinal cord injury: a systematic review and meta-analysis of incidence proportion and contributing factors. *Spinal Cord* 57 526–539. 10.1038/s41393-019-0274-4 30967602

[B62] Kapadia N. Masani K. Craven B. Giangregorio L. Hitzig S. Richards K. (2014). A randomized trial of functional electrical stimulation for walking in incomplete spinal cord injury: effects on walking competency. *J. Spinal Cord Med.* 37 511–524. 10.1179/2045772314Y.0000000263 25229735 PMC4166186

[B63] Kaski D. Dominguez R. Allum J. Bronstein A. (2013). Improving gait and balance in patients with leukoaraiosis using transcranial direct current stimulation and physical training: an exploratory study. *Neurorehabil. Neural Repair* 27 864–871. 10.1177/1545968313496328 23897903

[B64] Kaski D. Quadir S. Patel M. Yousif N. Bronstein A. (2012). Enhanced locomotor adaptation aftereffect in the “broken escalator” phenomenon using anodal tDCS. *J. Neurophysiol.* 107 2493–2505. 10.1152/jn.00223.2011 22323638 PMC3362242

[B65] Kim C. Eng J. Whittaker M. (2004). Level walking and ambulatory capacity in persons with incomplete spinal cord injury: relationships with muscle strength. *Spinal Cord* 42 156–162. 10.1038/sj.sc.3101569 15001980 PMC3226791

[B66] Kim T. Kim H. Wright D. (2021b). Improving consolidation by applying anodal transcranial direct current stimulation at primary motor cortex during repetitive practice. *Neurobiol. Learn. Mem.* 178:107365. 10.1016/j.nlm.2020.107365 33348047

[B67] Kim T. Buchanan J. Bernard J. Wright D. (2021a). Improving online and offline gain from repetitive practice using anodal tDCS at dorsal premotor cortex. *Npj Sci. Learn.* 6:31. 10.1038/s41539-021-00109-4 34686693 PMC8536655

[B68] Kinney A. Eakman A. Graham J. (2020). Novel effect size interpretation guidelines and an evaluation of statistical power in rehabilitation research. *Arch. Phys. Med. Rehabil.* 101 2219–2226. 10.1016/j.apmr.2020.02.017 32272106

[B69] Kressler J. Nash M. Burns P. Field-Fote E. (2013). Metabolic demand and muscle activation during different forms of bodyweight supported locomotion in men with incomplete SCI. *Arch. Phys. Med. Rehabil.* 94 1436–1442. 10.1155/2014/632765 23473703

[B70] Kumru H. Murillo N. Benito-Penalva J. Tormos J. (2016). Transcranial direct current stimulation is not effective in the motor strength and gait recovery following motor incomplete spinal cord injury during Lokomat gait training. *Neurosci. Lett.* 620 143–147. 10.1016/j.neulet.2016.03.056 27040426

[B71] Labruna L. Stark-Inbar A. Breska A. Dabit M. Vanderschelden B. Nitsche M. (2019). Individual differences in TMS sensitivity influence the efficacy of tDCS in facilitating sensorimotor adpatation. *Brain Stimul.* 12 992–1000. 10.1016/j.brs.2019.03.008 30930208 PMC6592723

[B72] Lakens D. (2013). Calculating and reporting effect sizes to facilitate cumulative science: a practical primer for t-tests and ANOVAs. *Front. Psychol.* 4:863. 10.3389/fpsyg.2013.00863 24324449 PMC3840331

[B73] Leech K. Hornby T. (2017). High-intensity locomotor exercise increases brain-derived neurotrophic factor in individuals with incomplete spinal cord injury. *J. Neurotrauma* 34 1240–1248. 10.1089/neu.2016.4532 27526567 PMC5359683

[B74] Leech K. Kinnaird C. Holleran C. Kahn J. Hornby T. (2016). Effects of locomotor exercise intensity on gait performance in individuals with incomplete spinal cord injury. *Phys. Ther.* 96 1919–1929. 10.2522/ptj.20150646 27313241 PMC5131185

[B75] Lemay J. Nadeau S. (2010). Standing balance assessment in ASIA D paraplegic and tetraplegic participants: construct validity of the Berg Balance Scale. *Spinal Core* 48 245–250. 10.1038/sc.2009.119 19773797

[B76] Lewek M. Sawicki G. (2019). Trailing limb angle is a surrogate for propulsive limb forces during walking post-stroke. *Clin. Biomech.* 67 115–118. 10.1016/j.clinbiomech.2019.05.011 31102839 PMC6635006

[B77] Lotter J. Henderson C. Plawecki A. Holthus M. Lucas E. Ardestani M. (2020). Task-specific vs impairment-based training on locomotor performance in individuals with chronic spinal cord injury: a randomized cross-over study. *Neurorehabil. Neural Repair* 34 627–639. 10.1177/1545968320927384 32476619 PMC7329565

[B78] Manella K. Roach K. Field-Fote E. (2013). Operant conditioning to increase ankle control or decrease reflex excitability improves reflex modulation and walking function in chronic spinal cord injury. *J. Neurophysiol.* 109 2666–2679. 10.1152/jn.01039.2011 23468393

[B79] Mang C. Brown K. Neva J. Snow N. Campbell K. Boyd L. (2016). Promoting motor cortical plasticity with acute aerobic exercise: a role for cerebellar circuits. *Neural Plasticity* 2016:6797928. 10.1155/2016/6797928 27127659 PMC4834415

[B80] Mang C. Campbell K. Ross C. Boyd L. (2013). Promoting neuroplasticity for motor rehabilitation after stroke: considering the effects of aerobic exercise and genetic variation on brain-derived neurotrophic factor. *Phys. Ther.* 93 1707–1716. 10.2522/ptj.20130053 23907078 PMC3870490

[B81] Mang C. Snow N. Campbell K. Ross C. Boyd L. (2014). A single bout of high-intensity aerobic exercise facilitates response to paired associative stimulation and promotes sequence-specific implicit motor learning. *J. Appl. Physiol.* 117 1325–1336. 10.1152/japplphysiol.00498.2014 25257866 PMC4254838

[B82] McDonnell M. Buckley J. Opie G. Ridding M. Semmler J. (2013). A single bout of aerobic exercise promotes motor cortical neuroplasticity. *J. Appl. Physiol.* 114 1174–1182. 10.1152/japplphysiol.01378.2012 23493367

[B83] McMillan D. Maher J. Jacobs K. Nash M. Gater D. (2021). Exercise interventions targeting obesity in persons with spinal cord injury. *Top. Spinal Cord Inj. Rehabil.* 27 109–120. 10.46292/sci20-00058 33814889 PMC7983638

[B84] McMorris T. Hale B. Corbett J. Robertson K. Hodgson C. (2015). Does acute exercise affect the performance of whole-body, psychomotor skills in an inverted-U fashion? A meta-analytic investigation. *Physiol. Behav.* 141 180–189. 10.1016/j.physbeh.2015.01.010 25582516

[B85] Merholz J. Kugler J. Pohl M. (2012). Locomotor training for walking after spinal cord injury. *Cochrane Database Syst. Rev.* 11 1465–1858. 10.1002/14651858.CD006676.pub3 PMC11848707 23152239

[B86] Meteyard L. Davies R. (2020). Best practice guidance for linear mixed-effects models in psychological science. *J. Mem. Lang.* 112:104092. 10.1016/j.jml.2020.104092

[B87] Milani P. Piu P. Popa T. della Volpe R. Bonifazi M. Rossi A. (2010). Cortisol-induced effects on human cortical excitability. *Brain Stimul.* 3 131–139. 10.1016/j.brs.2009.07.004 20633442

[B88] Miyazaki T. JKawada M. Nakai Y. Kiyama R. Yone K. (2019). Validity of measurement for trailing limb angle and propulsion force during gait using a magnetic intertial measurement unit. *BioMed Res. Int.* 2019:8123467. 10.1155/2019/8123467 31930138 PMC6942796

[B89] Monte-Silva K. Kuo M. Hessenthaler S. Fresnoza S. Liebetanz D. Paulus W. (2013). Induction of late LTP-like plasticity in the human motor cortex by repeated non-invasive brain stimulation. *Brain Stimul.* 6 424–432. 10.1016/j.brs.2012.04.011 22695026

[B90] Moore C. Carter R. Nietert P. Stewart P. (2011). Recommendations for planning pilot studies in clinical and translational research. *Clin. Transl. Sci.* 4 332–337. 10.1111/j.1752-8062.2011.00347.x 22029804 PMC3203750

[B91] Morrison S. Lorenz D. Eskay C. Forrest G. Basso M. (2018). Longitudinal recovery and reduced costs after 120 sessions of locomotor training for motor incomplete spinal cord injury. *Arch. Phys. Med. Rehabil.* 99 555–562. 10.1016/j.apmr.2017.10.003 29107040

[B92] Mutha P. Sainburg R. Haaland K. (2011). Critical neural substrates for correcting unexpected trajectory errors and learning from them. *Brain* 134 3647–3661. 10.1093/brain/awr275 22075071 PMC3235559

[B93] Neville B. Murray D. Rosen K. Bryson C. Collins J. Guccione A. (2019). Effects of performance-based training on gait and balance in individuals with incomplete spinal cord injury. *Arch. Phys. Med. Rehabil.* 100 1888–1893. 10.1016/j.apmr.2019.03.019 31026461

[B94] Nitsche M. Paulus W. (2000). Excitability changes induced in the human motor cortex by weak transcranial direct current stimulation. *J. Physiol.* 527 633–639. 10.1111/j.1469-7793.2000.t01-1-00633.x 10990547 PMC2270099

[B95] Nitsche M. Schauenburg A. Lang N. Liebetanz D. Exner C. Paulus W. (2003). Facilitation of implicit motor learning by weak transcranial direct current stimulation of the primary motor cortex in the human. *J. Cogn. Neurosci.* 15 619–626. 10.1162/089892903321662994 12803972

[B96] Nooijen C. Ter Hoeve N. Field-Fote E. (2009). Gait quality is improved by locomotor training in individuals with SCI regardless of training approach. *J. NeuroEng. Rehabil.* 6:36. 10.1186/1743-0003-6-36 19799783 PMC2764722

[B97] Olson R. Piercy K. Troiano R. Ballard R. Fulton J. Galuska D. (2018). *Physical Activity Guidelines For Americans*, 2nd Edn. Washington, DC: U.S. Department of Health and Human Services.

[B98] Page S. Cunningham D. Plow E. Blazack B. (2015). It takes two: non invasive brain stimulation combined with neurorhabilitation. *Arch. Phys. Med. Rehabil.* 96 S89–S93. 25813373 10.1016/j.apmr.2014.09.019 PMC4445084

[B99] Parker R. Weir C. (2020). Non-adjustment for multiplr testing in multi-arm trials of distinct treatments: rationale and justification. *Clin. Trials* 17 562–566. 10.1177/1740774520941419 32666813 PMC7534018

[B100] Pepin A. Ladouceur M. Barbeau H. (2003). Treadmill walking in incomplete spinal-cord-injured subjects: 2. Factors limiting the maximal speed. *Spinal Cord* 41 271–279. 10.1038/sj.sc.3101453 12714989

[B101] Peters D. Thibaudier Y. Deffeyes J. Baer G. Hayes H. Trumbower R. (2018). Constraints on stance-phase force production during overground walking in persons with chronic incomplete spinal cord injury. *J. Neurotrauma* 35 467–477. 10.1089/neu.2017.5146 28762876 PMC5793954

[B102] Peterson C. Cheng J. Kautz S. Neptune R. (2010). Leg extension is an important predictor of paretic leg propulsion in hemiparetic walking. *Gait Posture* 32 451–456. 10.1016/j.gaitpost.2010.06.014 20656492 PMC2974765

[B103] Peterson C. Kautz S. Neptune R. R. (2011). Braking and propulsive impulses increases with speed during accelerated and decelerated walking. *Gait Posture* 33 562–567. 10.1016/j.gaitpost.2011.01.010 21356590 PMC3085638

[B104] Peterson T. Willerslev-Olsen M. Conway B. Nielsen J. (2012). The motor cortex drives the muscles during walking in human subjects. *J. Physiol*. 10 2443–2452. 10.1113/jphysiol.2012.227397 22393252 PMC3424763

[B105] Phillips C. Baktir M. Srivatsan M. Salehi A. (2014). Neuroprotective effects of physical activity on the brain: a closer look at trophic factor signaling. *Front. Cell. Neurosci.* 8:170. 10.3389/fncel.2014.00170 24999318 PMC4064707

[B106] Potter-Baker K. Janini D. Lin Y. Sankarasubramanian V. Cunningham D. Varnerin N. (2018). Transcranial direct current stimulation (tDCS) paired with massed practice training to promote adaptive plasticity and motor recovery in chronic incomplete tetraplegia: a pilot study. *J. Spinal Cord Med.* 41 503–517. 10.1080/10790268.2017.1361562 28784042 PMC6117576

[B107] Raithatha R. Carrico C. Powell E. Westgate P. Chellette I. I. Lee K. (2016). Non-invasive brain stimulation and robot-assisted gait training after incomplete spinal cord injury: a randomized pilot study. *NeuroRehabilitation* 38 15–25. 10.3233/NRE-151291 26889794

[B108] Reis J. Schambra H. Cohen L. (2009). Noninvasive cortical stimulation enhances motor skill acquisition over multiple days through an effect on consolidation. *Proc. Natl. Acad. Sci.* 106 1590–1595. 10.1073/pnas.0805413106 19164589 PMC2635787

[B109] Robertson E. Pascual-Leone A. Miall R. (2004). Current concepts in procedural consolidation. *Nat. Rev.Neurosci.* 5 576–582. 10.1038/nrn1426 15208699

[B110] Roelker S. Bowden M. Kautz S. Neptune R. (2019). Paretic propulsion as a measure of walking performance and functional motor recovery post-stroke: a review. *Gait Posture* 68 6–14. 10.1016/j.gaitpost.2018.10.027 30408710 PMC6657344

[B111] Rojas Vega S. Abel T. Lindschulten R. Hollman W. Bloch W. Struder H. (2008). Impact of exercise on neuroplasticity-related proteins in spinal cord injured humans. *Neurosci. Biobehav. Rev.* 153 1064–1070. 10.1016/j.neuroscience.2008.03.037 18440711

[B112] Schmidt R. (1975). A schema theory of discrete motor skill learning. *Psychol. Rev.* 82 225–260. 10.1037/h0076770

[B113] Schubert M. Beck S. Taube W. Amtage F. Faist M. Gruber M. (2008). Balance training and ballistic strength training are associated with task-specific corticospinal adaptations. *Eur. J. Neurosci.* 27 2007–2018. 10.1111/j.1460-9568.2008.06186.x 18412622

[B114] Singh A. Staines W. (2015). The effects of acute aerobic exercise on the primary motor cortex. *J. Motor Behav.* 47 328–339. 10.1080/00222895.2014.983450 25565153

[B115] Singh A. Duncan R. Neva J. Staines W. (2014a). Aerobic exercise modulates intracortical inhibition and facilitation in a nonexercised upper limb muscle. *BMC Sports Sci. Med. Rehabil.* 6:23. 10.1186/2052-1847-6-23 25031838 PMC4100033

[B116] Singh A. Neva J. Staines W. (2014b). Acute exercise enhances the response to paired associative stimulation-induced plasticity in the primary motor cortex. *Exp. Brain Res.* 232 3675–3685. 10.1007/s00221-014-4049-z 25096384

[B117] Singh H. Chan K. Cheung L. Hitzig S. Musselman K. (2021). The impact of falls and fear of falling on participartion, autonomy, and life satisfaction among individuals with spinal cord injury: a brief report. *J. Spinal Cord Med.* 44 S234–S239. 10.1080/10790268.2021.1943251 34779724 PMC8604446

[B118] Skriver K. Roig M. Lundbye-Jensen J. Pingel J. Helge J. Kiens B. (2014). Acute exercise improves motor memory: exploring potential biomarkers. *Neurobiol. Learn. Mem.* 116 46–58. 10.1016/j.nlm.2014.08.004 25128877

[B119] Sohn W. Tan A. Hayes H. Pochiraju S. Deffeyes J. Trumbower R. (2018). Variability of leg kienmatics during overground walking in persons with chronic incomplete spinal cord injury. *J. Neurotrauma* 35 2519–2529. 10.1089/neu.2017.5538 29648987 PMC6205771

[B120] Sriraman A. Oishi T. Madhaven S. (2014). Timing-dependent priming effects of tDCS on ankle motor skill learning. *Brain Res.* 1581 23–29. 10.1016/j.brainres.2014.07.021 25063361 PMC4166556

[B121] Stevens S. Caputo J. Fuller D. Morgan D. (2015). Effects of underwater treadmill training on leg strength, balance, and walking performance in adults with incomplete spinal cord injury. *J. Spinal Cord Med.* 38 91–101. 10.1179/2045772314Y.0000000217 24969269 PMC4293539

[B122] Tepavac D. Field-Fote E. (2001). Vector coding: a technique for qunatification of intersegmental coupling in multicyclic behaviors. *J. Appl. Biomech.* 17 259–270. 10.1123/jab.17.3.259

[B123] Tschopp M. Sattelmayer M. Hilfiker R. (2011). Is power training or conventional resistance training better for function in elederly persons? A meta-analysis. *Age Ageing* 40 549–556. 10.1093/ageing/afr005 21383023

[B124] Tseng S. Chang S. Hoerth K. Nguyen A. Perales D. (2020). Anodal transcranial direct current stimulation enhances retention of visuomotor stepping skills in healthy adults. *Front. Hum. Neurosci.* 14:251. 10.3389/fnhum.2020.00251 32676018 PMC7333563

[B125] Van Cutsem M. Duchateua J. Hainaut K. (1998). Changes in single motor unit behaviour contribute to the increase in contraction speed after dynamic training in humans. *J. Physiol.* 1 295–305. 10.1111/j.1469-7793.1998.295by.x 9782179 PMC2231276

[B126] van Hedel H. Group E. S. (2009). Gait speed in relation to categories of functional ambulation after spinal cord injury. *Neurorehabil. Neural Repair* 23 343–350. 10.1177/1545968308324224 19036717

[B127] van Hedel H. Tomatis L. Muller R. (2006). Modulation of leg muscle activity and gait kinematics by walking speed and bodyweight unloading. *Gait Posture* 24, 35–45. 10.1016/j.gaitpost.2005.06.015 16099161

[B128] van Middendorp J. Hosman A. Donders A. (2011). A clinical prediction rule for ambulation outcomes after traumatic spinal cord injury: a longitudinal cohort study. *Lancet* 377 1004–1010. 10.1016/S0140-6736(10)62276-3 21377202

[B129] van Silfhout L. Hosman A. Bartels R. Edwards M. Abel R. Curt A. (2017). Ten meters walking speed in spinal cord-injured patients: does speed predict who walks and who rolls? *Neurorehabil. Neural Repair* 31 842–850. 10.1177/1545968317723751 28786305

[B130] Vietnen M. Welch C. (2020). The kinematics of cyclic human movement. *PLoS One* 15:e0225157. 10.1371/journal.pone.0225157 32134925 PMC7058299

[B131] Wanner P. Cheng F. Steib S. (2020). Effects of acute cardiovascular exercise on motor meoory encoding and cosnolidation: a systematic review and meta-analysis. *Neurosci. Biobehav. Rev.* 116 365–381. 10.1016/j.neubiorev.2020.06.018 32565171

[B132] Wilson J. Loenneke J. Jo E. Wilson G. Zourdos M. Kim J. (2012). The effects of endurance, strength, and power training on muscle fiber type shifting. *J. Strength Cond. Res.* 26 1724–1729. 10.1519/JSC.0b013e318234eb6f 21912291

[B133] Wirth B. van Hedel H. Curt A. (2008a). Ankle paresis in incomplete spinal cord injury: relation to corticospinal conductivity and ambulatory capacity. *J. Clin.Neurophysiol.* 25 210–217. 10.1097/WNP.0b013e318183f4e3 18677185

[B134] Wirth B. van Hedel H. Curt A. (2008b). Changes in corticopsinal function and ankle motor control during recovery from incomplete spinal cord injury. *J. Neurotrauma* 25 467–478. 10.1089/neu.2007.0472 18419251

[B135] Wirz M. Muller R. Bastiaenen C. (2010). Falls in persons with spinal cord injury: validity and reliability of the Berg Balance Scale. *Neurorehabil. Neural Repair* 24 70–77. 10.1177/1545968309341059 19675123

[B136] Wittkopf P. Larsen D. Graven-Neilsen T. (2021). Protocols for inducing homeostatic plasticity reflected in corticospinal exctiability in healthy human participants: a systematic review and meta-analysis. *Eur. J. Neurosci.* 54 5444–5461. 10.1111/ejn.15389 34251703

[B137] Yamaguchi T. Fujiwara T. Tsai Y. Tang S. Kawakami M. Mizuno K. (2016). The effects of anodal transcranial direct current stimulation and patterned electrical stimulation on spinal inhibitory interneruons and motor function in patients with spinal cord injury. *Exp. Brain Res.* 234 1469–1478. 10.1007/s00221-016-4561-4 26790423 PMC4851690

[B138] Yoshi A. Constantine-Paton M. (2009). Postsynaptic BDNF-TrkB signaling in synapse maturation, plasticity, and disease. *Dev. Neurobiol.* 70 304–322. 10.1002/dneu.20765 20186705 PMC2923204

[B139] Yozbatiran N. Keser Z. Davis M. Stampas A. O”Malley M. Cooper-Hay C. (2016). Transcranial direct current stimulation (tDCS) of the primary motor cortex and robot-assisted arm training in chronic incomplete cervical spinal cord injury: a proof of concept sham-randomized clinical study. *NeuroRehabilitation* 39 401–411. 10.3233/NRE-161371 27589510

