# Supplementary figures and images for: Walking and Balance Outcomes Are Improved Following Brief Intensive Locomotor Skill Training but Are Not Augmented by Transcranial Direct Current Stimulation in Persons With Chronic Spinal Cord Injury
Source: Front Hum Neurosci. 2022 May 11;16:849297. doi: 10.3389/fnhum.2022.849297 (PMC9130633; doi:10.3389/fnhum.2022.849297)

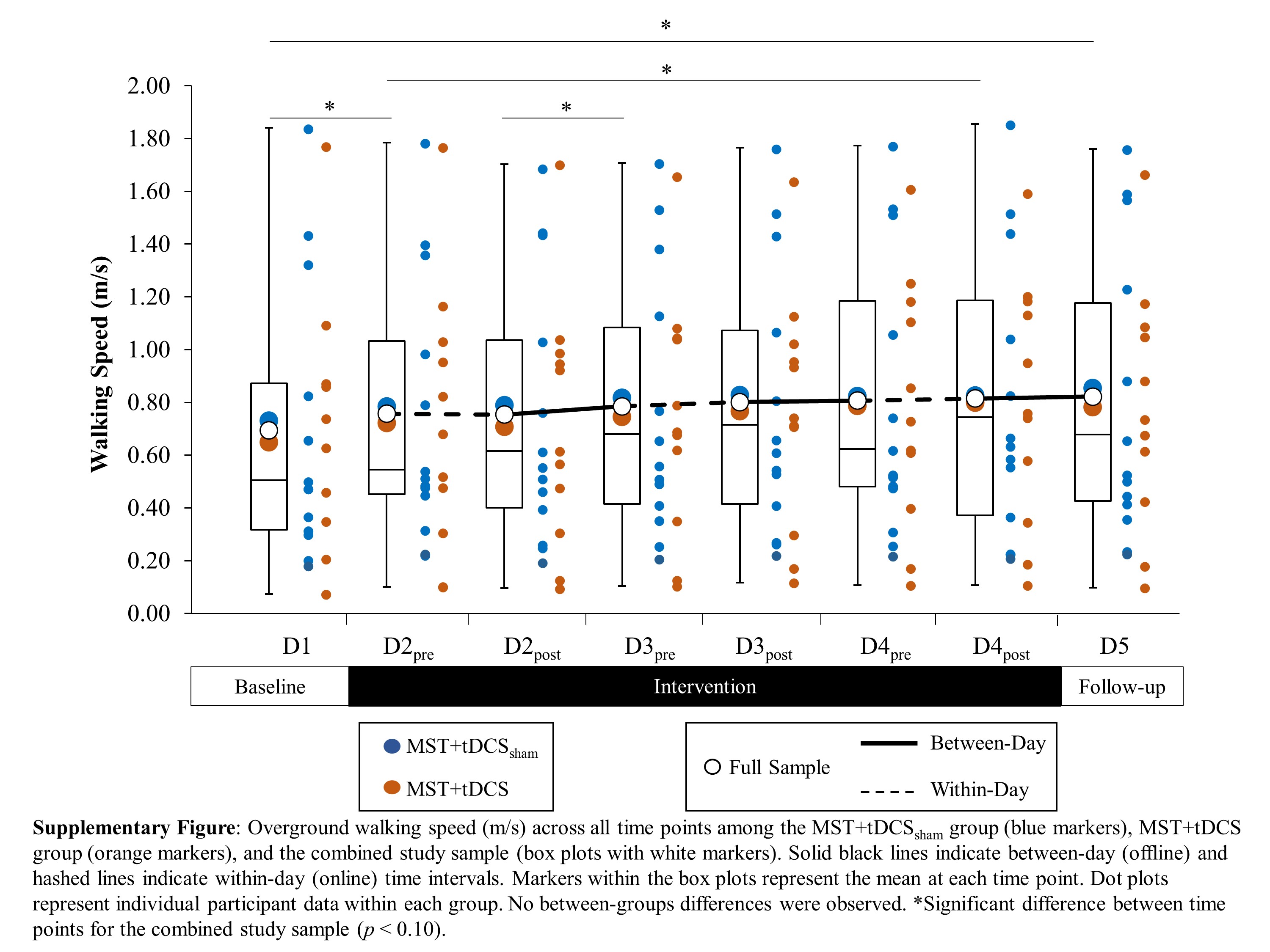

Supplement: Supplementary file 1 [file Image_1.jpg]
